# An unusual dual sugar-binding lectin domain controls the substrate specificity of a mucin-type O-glycosyltransferase

**DOI:** 10.1126/sciadv.adj8829

**Published:** 2024-02-28

**Authors:** Abbie M. Collette, Sergio A. Hassan, Susan I. Schmidt, Alexander J. Lara, Weiming Yang, Nadine L. Samara

**Affiliations:** ^1^ Structural Biochemistry Unit, NIDCR, NIH, Bethesda, MD 20892, USA.; ^2^ Bioinformatics and Computational Biosciences Branch, OCICB, NIAID, NIH, Bethesda, MD 20892, USA.; ^3^ MICaB Program, University of Minnesota Medical School, Minneapolis, MN 55455, USA.; ^4^ Section on Biological Chemistry, NIDCR, NIH, Bethesda, MD 20892, USA.

## Abstract

N-acetylgalactosaminyl-transferases (GalNAc-Ts) initiate mucin-type O-glycosylation, an abundant and complex posttranslational modification that regulates host-microbe interactions, tissue development, and metabolism. GalNAc-Ts contain a lectin domain consisting of three homologous repeats (α, β, and γ), where α and β can potentially interact with O-GalNAc on substrates to enhance activity toward a nearby acceptor Thr/Ser. The ubiquitous isoenzyme GalNAc-T1 modulates heart development, immunity, and SARS-CoV-2 infectivity, but its substrates are largely unknown. Here, we show that both α and β in GalNAc-T1 uniquely orchestrate the O-glycosylation of various glycopeptide substrates. The α repeat directs O-glycosylation to acceptor sites carboxyl-terminal to an existing GalNAc, while the β repeat directs O-glycosylation to amino-terminal sites. In addition, GalNAc-T1 incorporates α and β into various substrate binding modes to cooperatively increase the specificity toward an acceptor site located between two existing O-glycans. Our studies highlight a unique mechanism by which dual lectin repeats expand substrate specificity and provide crucial information for identifying the biological substrates of GalNAc-T1.

## Teaser

Cross-talk between two sugar-binding pockets of the O-glycosyltransferase GalNAc-T1 influences its complex substrate specificity.

## INTRODUCTION

Mucin-type O-glycosylation is a posttranslational modification that occurs on ~80% of proteins transported through the secretory pathway, influencing their structure, stability, and function (*1*). Substrates include the densely O-glycosylated mucin proteins responsible for the gel-like properties of mucus that lines and lubricates the epithelial surface to protect underlying layers from physical or chemical damage, prevent pathogen invasion, and maintain homeostasis through interactions with the microbiota. Aberrant mucin-type O-glycosylation is associated with various cancers, developmental disorders, neurological syndromes, metabolic diseases, and inflammation of the gastrointestinal tract (*2*–*5*).

**Fig. 1.**
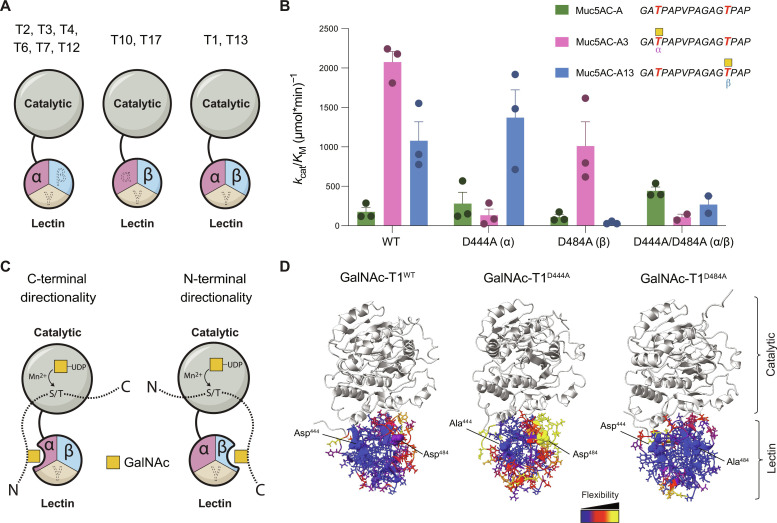
Characterization of the GalNAc-T1 lectin domain mutants. (**A**) GalNAc-T1 and its closest human homolog GalNAc-T13 are the only isoenzymes containing two lectin domain repeats (α and β) that can bind GalNAc. The catalytic domains are light gray, while the lectin domain repeats are shown in pink (α repeat), blue (β repeat), and beige (γ repeat). (**B**) Catalytic efficiency (*k*_cat_/*K*_M_) of GalNAc-T1^WT^, GalNAc-T1^D444A^, GalNAc-T1^D484A^, and GalNAc-T1^D444A/D484A^ against Muc5AC-A peptides. Putative acceptor sites on Muc5AC peptides are colored red. Yellow squares represent GalNAc. Assays were performed in triplicate in three independent experiments. Outliers were identified and eliminated using the ROUT method. Additional kinetic data are shown in fig. S1 and table S1. (**C**) Glycosylation N-terminal to a previous site is directed by the GalNAc-T1 β repeat, whereas the GalNAc-T1 α repeat dictates glycosylation in the C-terminal direction. (**D**) MD simulation results assessing side chain flexibility, one of the metrics used for comparative analysis of the dynamics upon single mutations in the α or β repeats of GalNAc-T1, showing the asymmetric changes introduced by the mutations compared to GalNAc-T1^WT^ (blue, <0.5 Å; yellow, >0.8 Å; red, in between). The starting model of human GalNAc-T1 was produced by AlphaFold2. The catalytic domain was excluded from the analysis.

A conserved family of N-acetylgalactosaminyl-transferases (GalNAc-Ts) initiate mucin-type O-glycosylation by catalyzing GalNAc transfer from uridine 5′-diphosphate (UDP)–GalNAc to a Thr/Ser on a protein to form Thr/Ser–O-GalNAc (*6*–*10*). GalNAc-Ts are Golgi-anchored type II membrane proteins containing two luminal domains that are important for activity, including an N-terminal Mn^2+^-dependent catalytic domain that adopts a glycosyltransferase family A (GT-A) fold (Fig. 1A). GalNAc-Ts contain a ricin B-type lectin domain that is connected to the C terminus of the catalytic domain via a ~10–amino acid linker. The lectin domain consists of three homologous repeats (α, β, and γ), where α and β, but not γ, have GalNAc-binding potential (*11*, *12*). Lectin domain binding to an extant GalNAc on a substrate (Thr–O-GalNAc) enhances the enzymatic activity of most GalNAc-Ts toward an acceptor site 7 to 11 amino acids away (*13*–*18*). For some GalNAc-Ts, the preferred acceptor site is N-terminal to the extant Thr–O-GalNAc (N-terminal directionality), and, for others, it is C-terminal (C-terminal directionality) (*15*, *16*).

In humans, 20 GalNAc-Ts with distinct and overlapping substrate specificities and functions meet the challenge of O-glycosylating numerous and diverse substrates (*19*). There is no consensus sequence for O-glycosylation. Instead, each isoenzyme follows its own rules for sequence recognition near an acceptor site (*20*–*22*). The isoenzyme GalNAc-T1 is highly expressed in most tissue types and influences many biological pathways, including salivary gland function, heart development, immune system function, and viral infectivity (*23*–*32*). However, the repertoire of substrates and the mechanisms by which GalNAc-T1 recognizes those substrates are poorly understood. GalNAc-T1 and its closest homolog GalNAc-T13 are the only human isoenzymes with a conserved GalNAc-binding motif consisting of Asp, His, and Asn in both the α and β repeats of the lectin domain (Fig. 1A and fig. S1A) (*33*). Previous studies show that GalNAc-T1 variants D444A (α) and D484A (β), but not D525A (γ), have reduced affinity toward glycosylated peptide and protein substrates, suggesting a role for both α and β in GalNAc-T1 activity (*34*, *35*). However, the mechanistic consequences of two GalNAc-binding lectin repeats and their role in substrate specificity have not been shown, and many of the biological substrates of GalNAc-T1 are still not known.

Here, we use biochemical, structural, and computational methods to gain insight into the mechanism of GalNAc-T1–mediated glycosylation and show that two lectin repeats influence GalNAc-T1 function and substrate specificity in multiple ways. First, we demonstrate that the α repeat directs O-glycosylation C-terminal to an existing Thr–O-GalNAc, while the β repeat directs O-glycosylation N-terminal to Thr–O-GalNAc. Unexpectedly, disrupting GalNAc binding in the α repeat influences the function of the β repeat and vice versa. We show that the effects on enzymatic activity are asymmetrical, with α and β affecting each other in distinct ways. Last, we show that the α and β repeats can interact with two Thr–O-GalNAc on a mucin 1 (Muc1) di-glycopeptide substrate through multiple binding modes to greatly enhance GalNAc-T1 activity toward an intermediate acceptor site. Overall, our results reveal the unique mechanisms of GalNAc-T1 lectin–mediated substrate recognition and expand our understanding of the diverse substrate specificities among this complex family of enzymes.

## RESULTS

### The GalNAc-T1 lectin domain α and β repeats have distinct directionality but function cooperatively

Studies using random peptide libraries show that GalNAc-T1 can efficiently glycosylate acceptor sites that are N- or C-terminal to a Thr–O-GalNAc (*16*); however, the molecular basis of this bidirectionality is not known. To investigate the roles of the GalNAc-T1 α and β repeats in glycopeptide substrate recognition and O-glycosylation directionality, we constructed lectin repeat variants to disrupt GalNAc binding in α (GalNAc-T1^D444A^), β (GalNAc-T1^D484A^) or both repeats (GalNAc-T1^D444A/D484A^). To simplify the kinetic analysis, we designed peptide and glycopeptide substrates based on the Muc5AC mucin repeat (GT***T***_***3***_PSPVPTTST***T***_***13***_SAP) but with only one or two acceptor sites (highlighted in bold): Muc5AC-A (GA***T***_***3***_PAPVPAGAG***T***_***13***_PAP); Muc5AC-A3 (GAT_3_^GalNAc^PAPVPAGAG***T***_***13***_PAP), for measuring glycosylation in the C-terminal direction; and Muc5AC-A13 (GA***T***_***3***_PAPVPAGAGT_13_^GalNAc^PAP) for measuring glycosylation in the N-terminal direction (Fig. 1B). On the basis of previous structural studies with Muc5AC peptides, we predict that the length of these peptides prevents them from concurrently binding both repeats, allowing the individual probing of each repeat with a single Thr–O-GalNAc (*36*). Using a bioluminescent glycosyltransferase assay (UDP-Glo, Promega), we measured the activity of wild-type (WT) and lectin domain variants of GalNAc-T1 against these substrates.

The *k*_cat_/*K*_M_ values show that GalNAc-T1^WT^ prefers glycosylated substrates Muc5AC-A3 and Muc5AC-A13 over the non-glycosylated peptide Muc5AC-A (Fig. 1B and fig. S1, B and C), consistent with previous studies showing that Thr–O-GalNAc enhances GalNAc-T1 activity via interactions with the lectin domain (*16*, *34*, *35*). Disrupting α repeat GalNAc binding (GalNAc-T1^D444A^) results in a 16-fold reduction in *k*_cat_/*K*_M_ compared to GalNAc-T1^WT^ when using Muc5AC-A3 as a substrate due to a 10-fold increase in *K*_M_ and 3-fold decrease in *k*_cat_ (Fig. 1B and fig. S1, B and C). These data suggest that both substrate binding and acceptor threonine alignment in the active site are affected by disrupting α repeat GalNAc binding. There are minimal changes to the activity of GalNAc-T1^D444A^ toward Muc5AC-A and Muc5AC-A13 compared to GalNAc-T1^WT^. Thus, the α repeat interacts with a Thr–O-GalNAc N-terminal to an acceptor site to specifically direct glycosylation in the C-terminal direction but does not affect O-glycosylation N-terminal to an existing Thr–O-GalNAc (Fig. 1C).

Disrupting β repeat GalNAc binding results in a 29-fold decrease in *k*_cat_/*K*_M_ for GalNAc-T1^D484A^ compared to GalNAc-T1^WT^ when using Muc5AC-A13 as a substrate, indicating that the β repeat has specificity toward a C-terminal Thr–O-GalNAc and directs glycosylation to an N-terminal acceptor site (Fig. 1, B and C). The decrease is driven by a 20-fold increase in *K*_M_ and 1.5-fold drop in *k*_cat_, suggesting that substrate binding has a more dominant role in influencing β repeat specificity than α repeat specificity (fig. S1C). Disrupting both the α and β repeats (GalNAc-T1^D444A/D484A^) results in a decrease in *k*_cat_/*K*_M_ for both mono-glycopeptides compared to GalNAc-T1^WT^, due primarily to an increase in *K*_M_ (Fig. 1B and fig. S1B), supporting earlier studies showing that glycopeptide specificity of GalNAc-T1 is dictated by the α and β repeats (*35*).

Unexpectedly, disrupting β repeat GalNAc binding (GalNAc-T1^D484A^) results in a twofold reduction in *k*_cat_/*K*_M_ compared to GalNAc-T1^WT^ when using Muc5AC-A3 as a substrate (Fig. 1B and fig. S1, B and C), indicating that the β repeat could also influence glycosylation C-terminal to a prior Thr–O-GalNAc. The reduction is due to a threefold decrease in *k*_cat_ with no substantial change to *K*_M_, indicating that correct substrate alignment, not enzyme-substrate complex formation, is affected by D484A. Thus, we hypothesize that a single–amino acid change in the β repeat could have long-range consequences on the function of the α repeat and that the two repeats function cooperatively. To assess how this cooperativity influences enzymatic function, we performed molecular dynamics (MD) simulations of GalNAc-T1^WT^, GalNAc-T1^D444A^ (α), GalNAc-T1^D484A^ (β), and GalNAc-T1^D444A/D484A^ (α and β) using apo-GalNAc-T1^WT^ as a template. Comparative analyses show that a mutation in one of the repeats induces side-chain reorientations and dynamic changes across the entire lectin domain with only minor backbone conformational changes, affecting the other repeat relative to GalNAc-T1^WT^ (Fig. 1D and fig. S2). The main differences are observed in side-chain flexibility and their long-range cross-correlation of movements. The observed effects on the β repeat when α repeat GalNAc binding is reduced (GalNAc-T1^D444A^) are distinct from the effects on the α repeat when the β repeat GalNAc binding is decreased (GalNAc-T1^D484A^), revealing asymmetrical cooperation between the repeats.

Modest but potentially notable H-bond rearrangements can also be seen at the lectin (β repeat)/catalytic domain interface that could influence the correct alignment of the substrate in the active site by stabilizing a realigned catalytic domain relative to the lectin domain (Fig. 1D and fig. S2). It is worth noting that loops 476-482 and 492-502 in the β repeat are closer to the catalytic domain than the corresponding loops in the α repeat and contain several basic and acidic residues (Asp^479^, Asp^480^, Lys^496^, His^498^, and His^499^) posed to interact with the catalytic domain; this is not the case for the corresponding loops in the α repeat, where only loop 446-454 contains charged residues (Arg^448^, Lys^449^, Glu^450^, Glu^452^, and Lys^453^) that are not well positioned to interact with the catalytic domain. Thus, any changes in their interaction patterns (H-bonds or salt bridges) are bound to have asymmetrical long-range consequences. These results suggest that disrupting one repeat even with a single mutation could trigger changes in the α/β, β/catalytic-domain, and peptide/lectin-domain interactions patterns, conceivably affecting *K*_M_ or *k*_cat_ independently, and explain the differences that we observed in the *k*_cat_ of Muc5AC-A3 between GalNAc-T1^WT^ and GalNAc-T1^D484A^ (fig. S1, B and C).

To verify that single point mutations in the α or β repeats prevent glycopeptide binding through direct interactions with Thr–O-GalNAc, we performed a GalNAc competition assay by measuring the activity of the GalNAc-T1 variants against the Muc5AC-A substrates in the presence of increasing concentrations of free GalNAc (fig. S3, A to D). We predict that high concentrations of free GalNAc could reduce GalNAc-T1 activity through two possible mechanisms: by replacing UDP-GalNAc in the active site and by interacting with lectin repeat GalNAc binding pockets to block glycopeptide substrate binding. The relative activity of GalNAc-T1^WT^ (relative to 0 mM free GalNAc) toward glycopeptides Muc5AC-A3 and Muc5AC-A13 decreases with increasing concentrations of free GalNAc, while the relative activity is not similarly perturbed toward the (unglycosylated) Muc5AC-A (fig. S3A). These observations demonstrate that GalNAc-T1^WT^ interacts directly with Thr–O-GalNAc via its lectin repeats. If a lectin repeat mutation prevents Thr–O-GalNAc binding, then free GalNAc should not be able to inhibit binding toward that substrate to the same extent as it does in GalNAc-T1^WT^. For GalNAc-T1^D444A^, the point mutation disrupts binding to Thr–O-GalNAc on Muc5AC-A3 but has minimal effect on interactions with Thr–O-GalNAc on Muc5AC-A13. Thus, free GalNAc does not compete with Muc5AC-A3 binding because the α repeat is disrupted but competes with Muc5AC-A13 binding because the β repeat is intact (fig. S3B). A similar rationale explains the results for GalNAc-T1^D484A^: Because the β repeat is altered and the α repeat is intact, we see unaffected activity toward Muc5AC-A13 and Muc5AC-A and greater inhibition toward Muc5AC-A3 in the presence of free GalNAc (fig. S3C). Free GalNAc does not affect the binding of GalNAc-T1^D444A/D484A^ to Muc5AC-A3 or Muc5AC-A13 to the same extent as GalNAc-T1^WT^, demonstrating that this variant binds GalNAc less efficiently through the lectin domain. Overall, these data support the kinetic data and show that a single–amino acid change in the α or β repeats can disrupt GalNAc binding.

### β repeat binding to a C-terminal Thr–O-GalNAc correctly positions an N-terminal Thr for catalysis

**Fig. 2.**
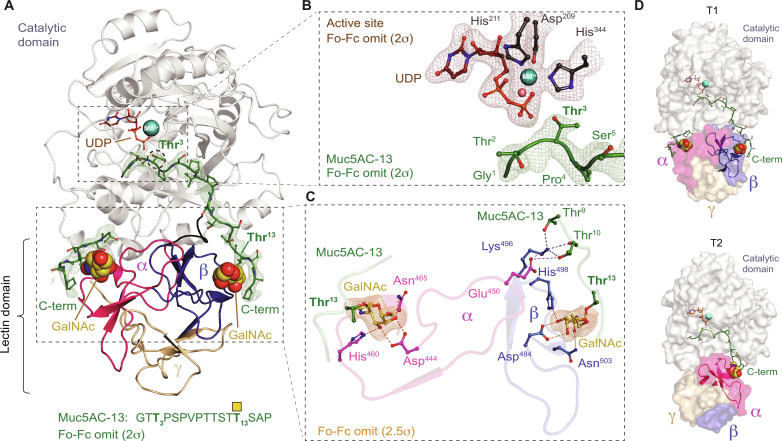
The x-ray crystal structure of GalNAc-T1. (**A**) The structure of GalNAc-T1 showing the catalytic (gray) and lectin domains in complex with UDP (brownish red), Mn^2+^ (cyan), and two Muc5AC-13 peptides (green) superimposed over a Fo-Fc omit map contoured at 2σ. GalNAc-T1 can interact with C-terminal Thr^13^–O-GalNAc (yellow sphere) using both its α (magenta) and β (blue) repeats but only the β repeat directs the acceptor Thr^3^ (red circle) into the active site. (**B**) A Fo-Fc omit map (brownish red) contoured at 2σ superimposed over the GalNAc-T1 active site containing UDP (brownish red), His^211^, Asp^209^, His^344^, and water that are coordinating a Mn^2+^ (cyan) with octahedral geometry. A Fo-Fc omit map contoured at 2σ (green) is superimposed over the N terminus of Muc5AC-13, showing the acceptor Thr^13^ correctly positioned in the active site for catalysis. (**C**) The α and β repeats of GalNAc-T1 showing GalNAc superimposed over a Fo-Fc omit map (orange) contoured at 2.5σ. Asp^444^, His^460^, and Asn^465^ are involved in hydrogen bonding to GalNAc in the α repeat, while residues Asp^484^, His^498^, and Asn^503^ participate in hydrogen bonds with GalNAc in the β repeat. β repeat GalNAc-T1 residue Lys^496^ makes hydrogen bonds with peptide residues Thr^9^ and Thr^10^ and a backbone carbonyl in β-bound Muc5AC-13. Glu^450^ in α repeat of GalNAc-T1 interacts with Lys^496^ in the β repeat and Thr^10^ in the β-bound Mu5AC-13, suggesting the presence of intra-lectin domain communications. (**D**) Comparing GalNAc-T1 in complex with Muc5AC-13, which uses the β repeat to direct glycosylation in the N-terminal direction to the crystal structure of GalNAc-T2 in complex with Muc5AC-13 (green), where Thr^13^–O-GalNAc (yellow sphere) binds to the α repeat (magenta) to direct glycosylation in the N-terminal direction [Protein Data Bank (PDB) ID: 5AJP] (*36*).

To further validate our biochemical data, we attempted to crystallize both human and mouse GalNAc-T1 (98% identity overall, and 99% identity in the lectin domain) with various glycopeptide substrates but were only able to obtain crystals and an x-ray crystal structure of mouse GalNAc-T1 in complex with Muc5AC-13, UDP, and Mn^2+^ (Muc5AC-13: GT***T***_***3***_PSPVPTTSTT_13_^GalNAc^SAP; table S2 and Fig. 2A) to 2.3-Å resolution. The asymmetric unit contains a dimer with one GalNAc-T1–Mn^2+^–UDP–(Muc5AC-13)_2_ complex (A) having well-resolved electron density and one GalNAc-T1–Mn^2+^–UDP–(Muc5AC-13)_1_ complex (B) with poor electron density. The analysis centers on complex A, where GalNAc-T1 is bound to two Muc5AC-13 glycopeptides, one via the α repeat and the other via the β repeat (Fig. 2A). The Muc5AC-13 glycopeptide interacting with the β repeat GalNAc binding pocket via Thr^13^–O-GalNAc correctly positions the acceptor Thr^3^ into the active site for catalysis (Fig. 2B). Thr^13^–O-GalNAc binding to GalNAc-T1-β adopts the same conformation as the conserved α repeat binding pocket, as shown by the structural alignment with GalNAc-T2-α (fig. S4A) (*36*–*39*). Unexpectedly, a second Muc5AC-13 peptide is bound to the α repeat via interactions with Thr–O-GalNAc13. However, the 2Fobs-Fcalc electron density for peptide residues beyond Thr^10^ is not clear and does not provide evidence that the α repeat correctly positions this glycopeptide into the active site for catalysis. Overall, the structure supports the biochemical data showing that the β repeat interacts with a C-terminal GalNAc on a glycopeptide to direct glycosylation in the N-terminal direction and correctly positions the acceptor Thr in the active site for catalysis.

Comparing apo-GalNAc-T1 (*40*) to substrate-bound GalNAc-T1 reveals that glycopeptide binding does not considerably alter lectin domain conformation, but subtle changes occur in the side chains of several residues, most notably in non-conserved residues Lys^496^ and Glu^450^ (figs. S1A and S4B). Lys^496^ adopts a distinct conformation and makes hydrogen bonds with the Thr^9^ and Thr^10^ side chains and a backbone carbonyl in Muc5AC-13 (Fig. 2C). Because other substrates do not necessarily contain Thr at similar positions, Lys^496^ binding to Muc5AC-13 could uniquely increase specificity toward this substrate via additional side-chain interactions, whereas the interaction with the backbone carbonyl is more likely to be conserved. MD simulations show that the Lys^496^ H-bonding patterns with residues in the α repeat and catalytic domain are influenced by mutations in the α and β repeats. Therefore, any intra-GalNAc-T1 weakening or strengthening of this residue’s H-bond is predicted to affect affinity and kinetics of peptide binding (fig. S2 and data S2). In addition, Glu^450^ in the α repeat interacts with Lys^496^ in the β repeat and Thr^10^ in the β-bound Muc5AC-13. These interactions can mediate communication between the repeats and further support the observations that Thr–O-GalNAc binding in the β repeat could have long-range effects on GalNAc binding in the α repeat and vice versa.

GalNAc-T2, which has one functional lectin repeat (α), can similarly glycosylate an acceptor site N-terminal to an existing GalNAc (*16*). We compared the structure of GalNAc-T1–Muc5AC-13 to GalNAc-T2–Muc5AC-13 to further understand how GalNAc-T1-β and GalNAc-T2-α can perform a similar function (*36*). The lectin domains of GalNAc-T1 and GalNAc-T2 have distinct orientations relative to their catalytic domain, positioning the β repeat of GalNAc-T1 in a similar location in space to the α repeat of GalNAc-T2 (Fig. 2D). Consequently, Thr^13^–O-GalNAc in Muc5AC-13 binds to the β repeat of GalNAc-T1 to position the N-terminal acceptor of the peptide in the active site similarly to α repeat binding of Muc5AC-13 in GalNAc-T2, showing how the two isoenzymes can O-glycosylate the same glycopeptide N-terminally from an extant GalNAc using distinct lectin repeats.

### The GalNAc-T1 lectin domain repeats cooperatively bind di-glycosylated Muc1

**Fig. 3.**
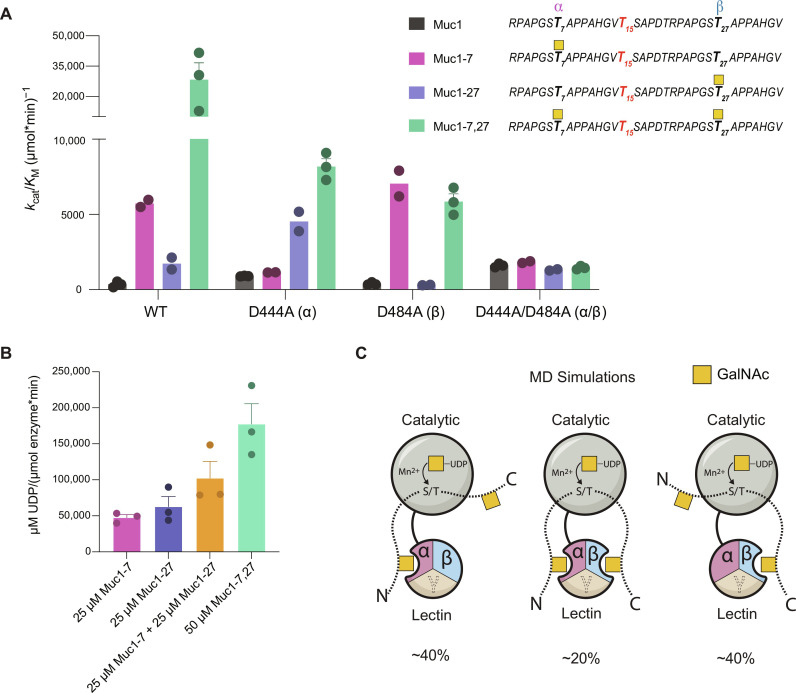
GalNAc-T1 lectin domain repeats synergistically bind diglycosylated Muc1. (**A**) Catalytic efficiency (*k*_cat_/*K*_M_) of GalNAc-T1^WT^, GalNAc-T1^D444A^, GalNAc-T1^D484A^, and GalNAc-T1^D444A/D484A^ against Muc1 peptides. GalNAc-T1 activity is synergistically enhanced by the presence of two GalNAcs and two active lectin repeats. Additional kinetic data are given in table S3 and fig. S6. (**B**) Activity assay verifying that the enhancement from the dual lectin domain GalNAc binding repeats of GalNAc-T1 is synergistic. Assays were performed in duplicate and replicated three times. (**C**) Schematic representation of the binding modes of diglycosylated Muc1 observed in the dynamics simulations. Stable binding of both GalNAcs was observed in some of the simulations (about 20% of the time; middle panel); in others, either the C terminus (40%, left) or the N terminus (40%, right) detached early on from the GalNAc-binding pocket, whereas the other GalNAc remained bound and stable. The intermediate Thr remained well positioned in the catalytic site in all cases (fig. S9, A and B).

Because the α and β repeats of GalNAc-T1 simultaneously bind Thr–O-GalNAc in the crystal structure, we reasoned that a longer glycosylated substrate could simultaneously interact with α and β via GalNAc to enhance the substrate specificity of GalNAc-T1 toward an intervening acceptor site. GalNAc-T1 glycosylation of the membrane-bound protein Muc1 is well characterized and occurs primarily at a specific Thr, making the kinetics easier to interpret (*41*–*45*). Therefore, we used Muc1 as our substrate to test the effect of simultaneous binding on substrate specificity. We designed a 34–amino acid Muc1 peptide containing one acceptor site for GalNAc-T1(bold) and two potential glycosylated sites that we predict will interact with both lectin repeats and correctly position the acceptor threonine -**T**SAP- into the active site (RPAPGS*T_7_*APPAHGV***T***_***15***_SAPDTRPAPGS*T_27_*APPAHGV; Fig. 3A for full panel). To verify O-glycosylation at Thr^15^, unglycosylated Muc1 treated with WT or variants of GalNAc-T1 was analyzed by higher-energy dissociation product ions-triggered electron-transfer/higher-energy collision dissociation (HCD-pd-EThcD) mass spectrometry followed by the software package pGlyco3 (*46*). Thr^15^ (***T***_***15***_SAP) was the only acceptor site identified with confidence on Muc1 after enzymatic treatment, consistent with previous results (fig. S5 and data S3). These data further show that lectin repeat mutations do not alter substrate specificity.

As observed with the Muc5AC-A peptides, the activity of GalNAc-T1^WT^ is enhanced by the presence of GalNAc on Muc1 due to decreases in *K*_M_ for all the Muc1 glycopeptide substrates compared to unglycosylated Muc1 (Fig. 3A and fig. S6, A and B). A single GalNAc at the N terminus (Muc1-7, predicted to bind the α repeat) shows a 16-fold increase in *k*_cat_/*K*_M_ compared to unglycosylated Muc1, while a GalNAc at the C terminus (Muc1-27, predicted to bind the β repeat) results in a 5-fold increase in *k*_cat_/*K*_M_ compared to unglycosylated Muc1 (Fig. 3A and table S3). For the di-glycopeptide (Muc1-7,27) containing GalNAc at the N and C termini, the *k*_cat_/*K*_M_ is 81-fold greater than for the unglycosylated peptide, suggesting that two GalNAcs on a substrate have a synergistic effect on the activity of GalNAc-T1^WT^. The increase in catalytic efficiency is driven by a decrease in *K*_M_, as *k*_cat_ is only slightly less than the *k*_cat_ for the unglycosylated peptide, indicating that, for Muc1-7,27, complex formation is the rate-limiting step and primary driver of substrate specificity and catalytic efficiency. Like GalNAc-T1, GalNAc-T2 is a bidirectional transferase, but with a single GalNAc binding lectin repeat (α) (*16*). The mechanism of GalNAc-T2 bidirectionality could be a function of its flexible linker that allows its lectin domain to adopt various conformations relative to the catalytic domain and accommodate variable glycopeptides (*16*, *36*, *47*). Unlike GalNAc-T1, GalNAc-T2 has similar *k*_cat_/*K*_M_ toward Muc1 mono- and di-glycopeptides (fig. S7 and table S4), providing evidence that the synergistic effect observed for GalNAc-T1 and Muc1 is unique and likely due to the presence of two GalNAc binding lectin repeats. We also observed a 1.5-fold difference in GalNAc-T1^WT^ activity toward 50 μM Muc1-7,27 in comparison to a combination mono-glycopeptides consisting of 25 μM Muc1-7 and 25 μM Muc1-27, indicating that dual binding contributes to the total activity and supporting the synergistic enhancement seen toward Muc1-7,27 in the kinetic data (Fig. 3B).

As predicted, a mutation in the α repeat (GalNAc-T1^D444A^) results in a fivefold decrease in *k*_cat_/*K*_M_ toward Muc1-7 compared to GalNAc-T1^WT^ that is driven by an increase in *K*_M_ (Fig. 3A; fig. S6, A and B; and table S3). A 2.5-fold increase in *k*_cat_/*K*_M_ toward Muc1-27 compared to GalNAc-T1^WT^ further indicates that mutations in one repeat can have global effects on enzyme function. While we expected to see similar activity for GalNAc-T1^D444A^ toward Muc1-27 and Muc1-7,27, the *k*_cat_/*K*_M_ of GalNAc-T1^D444A^ for the di-glycopeptide Muc1-7,27 is ~2-fold higher than Muc1-27 due to a lower *K*_M_ for the di-glycopeptide. This could be due to residual Thr–O-GalNAc binding to the α repeat pocket in the absence of Asp^444^, supported by a ~1.5-fold lower *K*_M_ for GalNAc-T1^D444A^ and Muc1-7 compared with unglycosylated Muc1 (Fig. 3A and fig. S6). The data do not indicate that residual binding of GalNAc occurs in the shorter Muc5AC-A3 peptide and GalNAc-T1^D444A^, suggesting that there may be additional interactions between amino acids in the longer Muc1 and GalNAc-T1 that help promote residual binding in the α repeat. Disrupting the β repeat in GalNAc-T1^D484A^ results in a 5.5-fold decrease in *k*_cat_/*K*_M_ toward Muc1-27 relative to GalNAc-T1^WT^ that is largely driven by a 10-fold reduction in the *k*_cat_. Thus, unlike the α repeat, a mutation in the β repeat is more likely influencing substrate alignment of Muc1-27 in the active site and catalytic turnover. This contrasts with GalNAc-T1^D484A^ activity toward Muc5AC-A13, where the decrease in *k*_cat_/*K*_M_ upon disruption of the β repeat is driven mainly by an increase in *K*_M_, indicating that peptide sequence, length, and flexibility could influence catalysis. In the case of Muc1-27, interactions with the peptide could have a more major compensatory role in the absence of GalNAc binding, in contrast to Muc5AC-A13. Lys^496^ or other residues could be interacting with Muc1-27 and compensating for the disruption of GalNAc binding via Asp^484^. Consequently, GalNAc could bind to the pocket due to proximity but not in a productive manner to properly align the acceptor Thr in the active site.

We used a GalNAc competition assay to further dissect Thr–O-GalNAc–mediated Muc1 glycopeptide binding to GalNAc-T1. Increasing concentrations of free GalNAc reduce the relative activity (relative to 0 mM GalNAc) of GalNAc-T1^WT^ toward the Muc1 glycopeptides compared with the unglycosylated Muc1 peptide (fig. S8A). Inhibition of GalNAc-T1^WT^ activity toward unglycosylated Muc1 is more extensive than observed for Muc5AC-A; however, this trend is consistent across variants, indicating that it does not appear to be affected by mutations in the α or β repeats (fig. S8, A to D). For GalNAc-T1^D444A^, increasing concentrations of free GalNAc do not decrease the relative activity toward Muc1-7 to the same extent as the other Muc1 substrates, suggesting that this mutation effectively reduces α repeat binding to Thr–O-GalNAc on Muc1-7 (fig. S8B). We expected to see an effect on Muc1-7,27 with GalNAc-T1^D444A^, but the data for Muc1-7,27 and Muc1-27 are similar, supporting the notion that a mutation in the α repeat has long-range effects on binding to the β repeat that, in this case, appear to compensate for the decreased α-mediated binding to Muc1-7,27.

For GalNAc-T1^D484A^ (β), we observe a decrease in the relative activity toward Muc1-27 at higher concentrations of free GalNAc than what is observed for the other substrates, showing that this mutation also prevents Thr–O-GalNAc from efficiently binding the β repeat (fig. S8C). Like GalNAc-T1^D444A^, a mutation in the β repeat does not seem to effect GalNAc mediated Muc1-7,27 binding, pointing to a compensatory effect by the unaltered α repeat. The relative activity of GalNAc-T1^D444A/D484A^ toward the Muc1 glycopeptides is greater than GalNAc-T1^WT^–mediated activity in our GalNAc competition assay, supporting a role for both repeats in Muc1 binding and glycosylation (fig. S8D). Together, the data suggest that disrupting one repeat leads to a compensatory role for the other repeat. Both the GalNAc-T1^D444A^ and GalNAc-T1^D484A^ variants show no major changes in relative activity in our GalNAc competition assay toward Muc1-7,27, providing evidence for intra-lectin domain compensation or influence. The experiments and simulations suggest that the dynamic changes in the β repeat (increased flexibility and decreased cross-correlations) are responsible for the enhanced enzymatic activity of GalNAc-T1^D444A^, whereas the opposite changes observed in the α repeat diminish the activity of GalNAc-T1^D484A^ and that these observations can be substrate specific. The changes in the H-bond network at the lectin/catalytic domain interface involving charged residues Asp^479^, Asp^480^, and Lys^496^, all in the β repeat, may also play a role, as they are also located at the peptide/enzyme interface.

To gain insight into the molecular and kinetic mechanisms of GalNAc-T1-Muc1 complex formation, we performed a series of MD simulations of the Muc1 di-glycopeptide bound to GalNAc-T1. The simulations suggest three main stable binding modes (Fig. 3C and fig. S9, A and B). The di-glycopeptide can bind to both repeats simultaneously, with hydrophobic and H-bond interactions stabilizing the two GalNAc in their respective binding sites. However, in most simulations, one GalNAc detaches from its pocket, whereas the other GalNAc remains bound and stable. Although there are noteworthy structural fluctuations of the bound peptides, in all the modes, the intermediate acceptor Thr tends to remain close to and correctly positioned in the catalytic site. The simulations support a kinetic model in which binding to either the α, β, or both repeats direct glycosylation (fig. S9, C and D), and simultaneous binding to both GalNAc moieties is not strictly necessary to increase specificity toward an intermediate acceptor site.

## DISCUSSION

GalNAc-T1 is a ubiquitous mucin-type O-glycosyltransferase that regulates critical cellular processes, including immunity and development. However, its substrate repertoire is not well characterized, and how GalNAc-T1–mediated O-glycosylation affects many of these processes is still not known. Like many of the isoenzymes in this family, GalNAc-T1 contains a conserved pocket in the active site that interacts with a proline residue three amino acids downstream to an acceptor site (+3 position) to enhance substrate binding and help align the acceptor Thr for catalysis, as seen in Muc5AC (-**T**_**3**_PS*P*- and -**T**_**13**_SA*P*-) and Muc1 (-**T**SA*P-*) (*47*). This gives GalNAc-T1 the ability to modify sites on unglycosylated substrates, such as the severe acute respiratory syndrome coronavirus (SARS) 2 spike protein, where O-glycosylation of Thr^678^ located near the frequently mutated Pro^681^ adjacent to the furin cleavage site (-**T**_**678**_NS*P*RRAR-) decreases the furin-mediated cleavage of spike (*31*, *32*). Although GalNAc-T1 can modify nascent peptides, both the α and β repeats in the lectin domain of GalNAc-T1 were shown to enhance enzymatic activity, indicating that GalNAc-T1 prefers glycopeptide substrates (*34*, *35*). However, the molecular details of how these repeats influence catalysis and substrate specificity have yet to be described.

**Fig. 4.**
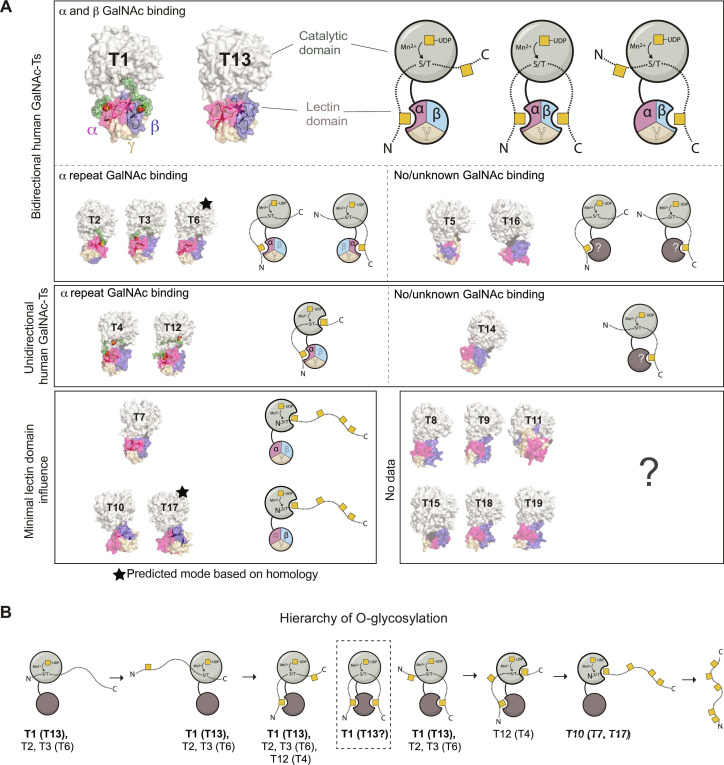
Lectin domains differentially influence the directionality of human GalNAc-Ts. (**A**) GalNAc-T1 and GalNAc-T13 are bidirectional due to two GalNAc-binding lectin repeats, and GalNAc-T1 (and likely GalNAc-T13) simultaneously binds two Thr–O-GalNAc via its α and β repeats. GalNAc-T2 and GalNAc-T3 (and possibly GalNAc-T6) are bidirectional but have only one GalNAc-binding lectin repeat (α). GalNAc-T5 and GalNAc-T16 are bidirectional but have no known ability to bind GalNAc via the lectin repeat. The α repeats of GalNAc-T4 and GalNAc-12 direct C-terminal glycosylation from a prior site with additional enhancement coming from catalytic domain GalNAc binding. GalNAc-T14 has N-terminal directionality but no evidence of lectin domain-mediated GalNAc binding. GalNAc-T7 and GalNAc-10 (and possibly GalNAc-T17) have GalNAc binding capability in the lectin domain (α for T7 and β for T10 and T17), but activity enhancement is driven by catalytic domain GalNAc binding. There are no data for GalNAc-T8, GalNAc-T9, GalNAc-T11, GalNAc-T15, GalNAc-T18, and GalNAc-T19. Structural models of human GalNAc-T3, GalNAc-T5, GalNAc-T6, GalNAc-T8, GalNAc-T9, GalNAc-T11, GalNAc-T13, GalNAc-T14, GalNAc-T15, GalNAc-T16, GalNAc-T17, GalNAc-T18, and GalNAc-T19 were generated with AlphaFold2 (*58*), and experimental structures were used for GalNAc-T1 (this paper); GalNAc-T2, GalNAc-T4, GalNAc-T7, GalNAc-T10, and GalNAc-T12 (*36*, *37*, *49*, *50*, *59*); and the peptide bound to human GalNAc-T3 (green) (*38*). Catalytic domains are shown in light gray, α in magenta, β in blue, γ in wheat, and peptides from experimental structures in green. (**B**) The hierarchal model for O-glycosylation. GalNAc-T1 was previously characterized as polypeptide preferring, early transferase. However, its lectin domain-mediated preference for glycopeptides suggests that it can function early in the process and at intermediate stages to efficiently glycosylate acceptor sites that are positioned between two prior sites.

In this study, we show that GalNAc-T1-α and GalNAc-T1-β cooperate to efficiently glycosylate substrates: The α repeat promotes glycosylation C-terminal to an extant GalNAc, while the β repeat supports N-terminal O-glycosylation to an extant GalNAc. In addition, we show that GalNAc-T1 activity is synergistically enhanced by the presence of two GalNAc groups on a di-glycopeptide substrate that can interact with both the α and β repeats of the lectin domain to correctly position an intermediate acceptor site for catalysis. The effect of mutations in α and β on GalNAc-T1 activity depends on the sequence, length, and flexibility of the substrate, as shown by the contrasting effects of lectin domain mutations on glycopeptides Muc1 and Muc5AC-A. The various substrate binding modes show how GalNAc-T1 can modify a broad substrate repertoire and influence diverse cellular pathways. The mechanism described for GalNAc-T1 can be extended to its closest homolog, GalNAc-T13, which is ~84% identical and has previously been shown to be bidirectional (Fig. 4A) (*16*). The two paralogs have virtually identical substrate specificity and are essentially regulated by their distinct cell expression patterns (*33*); GalNAc-T1 is more widely expressed, whereas GalNAc-T13 expression primarily occurs in neuronal cells (*48*). Understanding the mechanism of GalNAc-T13 glycosylation is likewise critical because *GALNT13* is associated with many cancers (*33*), further highlighting the importance of this study.

Apart from GalNAc-T13, these analyses cannot be generalized to other human GalNAc-Ts because the sequence identity between the lectin domain of GalNAc-T1 and the other isoenzymes is ~20 to 30%. This strongly indicates an immense degree of heterogeneity in how these repeats influence substrate binding, align the acceptor in the active site, and regulate enzyme function. GalNAc-T2, GalNAc-T3, GalNAc-T6, and GalNAc-T7 contain GalNAc binding pockets in the α repeat (Fig. 4A and fig. S1A). Both GalNAc-T2 and GalNAc-T3 are capable of glycosylating in both directions (*16*), and, on the basis of sequence homology, GalNAc-T6 would be expected to behave similarly to GalNAc-T3. For GalNAc-T2, bidirectionality could be due to the flexible linker between the catalytic and lectin domains, which could reorient the α repeat to promote binding to Thr–O-GalNAc with N- or C-terminal acceptor sites located 7 to 11 amino acids from the extant site (*36*, *39*). GalNAc-T12 and GalNAc-T4 have a strong preference for α repeat–mediated C-terminal O-glycosylation and contain GalNAc-binding pockets in their catalytic domains that further enhance enzymatic activity (*37*, *49*).

α Repeat enhancement is minimal for GalNAc-T7, which also contains a putative GalNAc binding pocket in the catalytic domain (*16*). GalNAc-T10 and, by extension, GalNAc-17 have GalNAc-binding residues in the β repeat and primarily exhibit GalNAc binding ability via the catalytic domain. Like GalNAc-T7, lectin domain enhancement is present but reduced for GalNAc-10 in comparison to other isoenzymes toward glycopeptides with lectin domain binding Thr–O-GalNAc sites. A chimeric protein consisting of GalNAc-T10 with a GalNAc-T1 linker increases enzymatic activity compared to GalNAc-T10^WT^, suggesting that the GalNAc-T10 linker may be preventing efficient lectin domain binding to a glycopeptide substrate (*50*). In contrast, GalNAc-T14 glycosylates N-terminal to a prior site on glycopeptides with lectin domain binding Thr–O-GalNAc sites, despite the lack of conserved GalNAc binding motifs within the lectin domain, and, similarly, GalNAc-T5 and GalNAc-T16 do not have GalNAc binding residues within the lectin domain but appear to be bidirectional (*16*). It is possible that the lectin domains of GalNAc-T14, GalNAc-T5, and GalNAc-T16 contain unidentified, distinct GalNAc binding residues or pockets, which remains to be determined. There are now no data on the lectin domains of GalNAc-T11, GalNAc-T15, or the Y family of transferases GalNAc-T8, GalNAc-T9, GalNAc-T18 and GalNAc-T19. Thus, many unknowns remain encoded within the lectin domains of many GalNAc-Ts, and studies on individual enzymes will provide insight into the unique roles of each distinct lectin domain.

The proposed sequential and hierarchal model of O-glycosylation starts with GalNAc-Ts that readily glycosylate nascent proteins (early transferases), followed by transferases that glycosylate partially glycosylated proteins (intermediate transferases), and concludes with GalNAc-Ts that recognize and further modify densely glycosylated substrates (late transferases) (Fig. 4B). The ability of GalNAc-T1 to glycosylate nascent peptides led to its characterization as “peptide-preferring” enzyme that functions as an early transferase (*51*, *52*). However, our data show that, although GalNAc-T1 is active against nascent peptides, its activity is synergistically amplified toward di-glycopeptides that can simultaneously interact via GalNAc to the α and β repeats. Thus, GalNAc-T1 is “peptide capable” as opposed to peptide preferring. This contrasts with strict glycopeptide-preferring isoenzymes like GalNAc-T10, GalNAc-T7, GalNAc-T4 and GalNAc-T12, which do not modify nascent peptides. Strict glycopeptide-preferring GalNAc-Ts typically contain a GalNAc binding pocket in their catalytic domain that allows them to glycosylate one to three amino acids away from the previous site (*16*, *37*). The data highlight a complex role for GalNAc-T1, where it functions at early and intermediate points in the glycosylation pathway depending on the substrate and the context (Fig. 4B).

Overall, this study provides an additional example of how each member of this large enzyme family, which does not recognize a consensus sequence or motif, follows its own rules for substrate recognition and activity. In doing so, the enzymes can work together to O-glycosylate a wide range of substrates. Some enzymes, like GalNAc-T1 and GalNAc-T2, function at multiple stages of O-glycosylation. Other enzymes have limited variability and acceptor site specificity. Each isoenzyme expressed within a cell type coordinates with other isoenzymes to correctly glycosylate the many proteins that will be secreted into the extracellular space. The location(s) of each individual enzyme within the Golgi adds another layer of regulation. However, it is not clear how relative GalNAc-T localization, aqueous environment, and molecular crowding influence GalNAc-T function in vivo.

Because many of the biological substrates of GalNAc-T1 have not been identified, how the lectin repeats influence site specificity in cells is still not known. We interrogated the National Cancer Institute Genomic Data Commons Data Portal and found four cases of bronchus/lung cancer associated with a D444H mutation in *GALNT1*, which is the most frequent cancer-associated mutation in the functional region of the enzyme and a case of bladder cancer associated with a D484N mutation. However, how mutations in *GALNT1* disrupt other biological pathways remains unclear. Thus, understanding the different ways that GalNAc-T1 O-glycosylates its substrates in vitro can be used to correctly interpret glycoproteomics data from human samples, particularly samples with mutations in *GALNT1*, and hopefully begin to understand the biological substrates that are affected by GalNAc-T1–mediated O-glycosylation in health and disease.

## MATERIALS AND METHODS

### Expression and purification of human and mouse GalNAc-T1

A glycerol stock of *Pichia pastoris* expressing mouse GalNAc-T1 (pKN55-His6-TEV-mGalNAc-T1-∆56) and a plasmid expressing human GalNAc-T1 (pKN55-His6-TEV-hGalNAc-T1-∆44) were gifts from the Tabak lab at the National Institute of Dental and Craniofacial Research (NIDCR) at the National Institutes of Health (NIH). GalNAc-T1^D444A^ and GalNAc-T1^D484A^ were generated by site-directed mutagenesis using pKN55-His6-TEV-hGalNAc-T1-∆44 as a template, and GalNAc-T1^D444A/D484A^ was generated by site-directed mutagenesis using GalNAc-T1^D444A^ as a template (table S5). The mutations were verified by DNA sequencing. Plasmids were isolated and linearized by Pmel and transformed by electroporation into SMD1168 *P. pastoris* expression cells (Invitrogen) to generate stable GalNAc-T1–expressing strains. Transformants were selected on Minimal Dextrose (MD) plates [1.34% (w/v) Yeast Nitrogen Base (YNB), 4× 10 to 5% (w/v) biotin, and 2% (w/v) dextrose]. All proteins were expressed and purified using the same protocol. Cultures of 1 liter were grown, and protein expression was induced and purified as described (*36*).

### Transferase assays and enzyme kinetics

Peptides and glycopeptides were synthesized and purified by Anaspec. Enzyme kinetics and activity assays were determined using the UDP-Glo Glycotransferase Assay (Promega). GalNAc-T1 and GalNAc-T2 glycosylation reactions (25 μl) were performed in a master mix containing 25 mM Hepes, 5 mM MnCl_2_, 100 mM NaCl, 5 mM β-mercaptoethanol (βME), and 25 μM ultra-pure UDP-GalNAc (Promega) (pH 7.3) in the presence of variable concentrations of peptide to generate enzyme kinetic curves. To initiate reactions against peptides Muc5AC-A, Muc5AC-A3, and Muc5AC-A13, 1 μl of ~0.3 μM WT human GalNAc-T1 or variants was added directly to the master mix and incubated at room temperature for 30 min. To initiate glycosylation reactions against the Muc1 set of peptides, the master mix was added to the 96-well plate containing 1 μl of ~0.4 μM GalNAc-T1 or ~1 nM GalNAc-T2. The reactions were then incubated at 37°C for 15 min. All reactions were stopped using 25 μl of UDP-detection reagent and mixed for 30 s in the double orbital mode at 365 cpm. Glycosylation reactions against peptides Muc5AC-A, Muc5AC-A3, and Muc5AC-A13 were then incubated for 1 hour at room temperature. Glycosylation reactions against the Muc1 set of peptides were incubated for 1 hour at exactly 27°C in the dark. In the case that bubbles were present following incubation, the 96-well plate was spun at 1600 rpm for 30 s. Luminescence values were obtained using a Synergy Neo2 (Biotek) with a 0.3-s integration time and automatic gain determination. To correlate the UDP concentration to luminescence and approximate the product formation, we generated a UDP standard curve in each assay consisting of 25 mM Hepes, 5 mM MnCl_2_, 100 mM NaCl, 5 mM βME, and 0 to 25 μM UDP (pH 7.3). The resulting kinetic values were corrected for free UDP-GalNAc hydrolysis before being fit to a Michaelis-Menten or substrate inhibition program in GraphPad Prism 9 software. The *K*_M_ and maximum velocity (*V*_max_) values were obtained from each biological replicate and used to calculate *k*_cat_ and *k*_cat_/*K*_M._ SEs were calculated where appropriate. The ROUT (robust regression and outlier removal) method was used to identify and eliminate outliers. Reactions were performed in duplicate or triplicate and repeated two to three times as noted. Both inhibition and activity assays were carried out as outlined above. GalNAc inhibition reactions were performed using 50 μM peptide substrate.

### Crystallization and data collection

Around 5 to 7 ml of purified mouse GalNAc-T1 (98% identity to human GalNAc-T1) stored at −80°C was thawed and buffer exchanged into 100 mM NaCl, 20 mM Hepes, 0.25 mM EDTA, and 10 mM βME (pH 7.3) by purification over a SD200 16/60 gel filtration column. Peak fractions were collected and concentrated to ~8 to 10 mg/ml using a 10,000-kDa cutoff spin column (Millipore). The complex was assembled by combining GalNAc-T1 with 4 mM Muc5AC-13 (Anaspec), 5 mM UDP, and 5 mM MnCl_2_ to a final GalNAc-T1 concentration of 5 mg/ml. The complex was incubated at room temperature for 15 min and then placed on ice. Hanging drops were set up by mixing 1 μl of complex with an equal volume of well buffer containing 20% PEG-3350 (polyethylene glycol, molecular weight 3350) and 0.1 M MES (pH 6.5). Crystals were cryoprotected with 20% glycerol, 20% PEG-3350, and 0.1 M MES (pH 6.5) and frozen with LN2. X-ray data were collected at the Advanced Photon Source SER-CAT BM-22 beam line (Argonne, IL). Data were processed and scaled using HKL2000 (*53*), and the initial structure was solved by Molecular Replacement (MolRep, CCP4i) (*54*, *55*) using the structure of apo-mGalNAc-T1 as a search model [Protein Data Bank (PDB) ID: 1XHB]. The structure was refined in PHENIX (*56*) and Coot (*57*) to 2.3-Å resolution, and figures were generated in PyMOL (The PyMOL Molecular Graphics System, version 2.0, Schrodinger LLC).

### Modeling and simulations

A model of the full-length human GalNAc-T1 (UniProt ID: Q10472) was built with AlphaFold2 (*58*). The poorly predicted 1-48 segment was removed, and the protein (apo-GalNAc-T1^WT^) was subjected to a 5-ns MD simulation at 37°C, 1 atm, 120 mM of NaCl, and pH 7 (Supplementary Materials and Methods). A conformation at the end of the simulation was used to build two complexes, one with UDP (UDP–apo-GalNAc-T1^WT^) and the other with UDP-GalNAc (UDP-GalNAc–apo-GalNAc-T1^WT^), along with Mn^2+^ ions, as observed in the crystal structures. Each complex was subjected to a 5-ns MD simulation under the above conditions. A conformation at the end of each of these simulations was used to build two additional complex with di-glycosylated human Muc1-derived peptide (repeat APGSTAPPAHGVTSAPDTRPAPGSTAPPA; with acetylated and amidated termini): UDP–apo-GalNAc-T1^WT^:Muc1 and UDP-GalNAc–UDP–apo-GalNAc-T1^WT^:Muc1-5-25, where Muc1-5-25 has α-D-GalNAc-L-Thr at positions 5 and 25 (the equivalent to Muc1-7-27; Fig. 3A). In building these protein:peptide complexes, two initial conformations of the peptide were considered: one obtained after a 1-ns MD simulation starting from a stretched backbone conformation and the other as modeled by AlphaFold in the context of the full-length hMuc1 (UniProt ID: 15941), predicted to be part of a solenoid horseshoe structure. The corresponding complexes were modeled through steered MD to position Thr^5^-GalNAc, Thr^13^, and Thr^25^-GalNAc close to their relative positions in the available crystal structures. The results reported here were practically the same regardless of the initial peptide conformation. The final complexes were subjected to (unbiased) 30-ns MD simulations at the above thermodynamic conditions, and all the analyses were carried out over the last 20 ns (Supplementary Materials and Methods). Ten independent simulations were performed for each complex.

## Supplementary Materials

### This PDF file includes:

Supplementary Materials and Methods Figs. S1 to S9 Tables S1 to S5 Legends for data S1 to S3 References
