# Supplementary material for: An unusual dual sugar-binding lectin domain controls the substrate specificity of a mucin-type O-glycosyltransferase
Source: Sci Adv. 2024 Feb 28;10(9):eadj8829. doi: 10.1126/sciadv.adj8829 (PMC10901373; doi:10.1126/sciadv.adj8829)
Supplement: Supplementary file 1 — Supplementary Materials and Methods Figs. S1 to S9 Tables S1 to S5 Legends for data S1 to S3 References [file sciadv.adj8829_sm.pdf]

Science Advances
AAAS

Supplementary Materials for

An unusual dual sugar-binding lectin domain controls the substrate specificity of a mucin-type O-glycosyltransferase

Abbie M. Collette *et al.*

Corresponding author: Nadine L. Samara, nadine.samara@nih.gov

Sci. Adv. **10**, eadj8829 (2024)
DOI: 10.1126/sciadv.adj8829

The PDF file includes:

Supplementary Materials and Methods
Figs. S1 to S9
Tables S1 to S5
Legends for data S1 to S3
References

Other Supplementary Material for this manuscript includes the following:

Data S1 to S3

Supplementary Text

Materials and Methods

Simulations setup

All the simulations were carried out in a cubic cell with cubic periodic boundary conditions using particle-mesh Ewald summations with the all-atom CHARMM *(61)* (param36) force field. The side length of the simulation box was initially set at ~12.0 nm and filled with ~56,000 TIP3P water molecules, yielding an average density of ~0.993 g/cm^3 after equilibration. In all the complexes, disulfide bonds Cys106-Cys339 and Cys330-Cys408 in the catalytic domain and Cys442-Cys459, Cys482-Cys497, and Cys523-Cys540 in the lectin domain were set at the beginning of the simulations. Protonation states were assigned based on pKa prediction with the PROPKA program (version 3.0) *(62, 63)* on the initial structure, all Asp and Glu were negatively charged, and Arg and Lys positively charged; His residues were generally neutral. Parameters for α-D-GalNAc-L-Threonine were created by chemical analogy from similar molecules in the CHARMM parameter files. The Na^+ and Cl^- ions were randomly distributed by replacing water molecules. All bond lengths involving hydrogen atoms were constrained with the SHAKE algorithm, and an integration step of 2 fs was used. The temperature and pressure were maintained with the Hoover thermostat, using a mass of 10^3 kcal mol^{-1}ps^2, and with the Langevin piston method, with mass and collision frequency of 400 amu and 20 ps^{-1}.

The corresponding protein:peptide complexes were modeled through steered MD in a continuum solvent model *(64, 65)* by applying a gradually increasing harmonic force to the heavy atoms of Thr5-O-GalNAc, Thr13, and Thr25-O-GalNac to bring them close to their relative positions observed in the available crystal structures: Thr5-GalNAc and Thr25-GalNAc near Asp444 and Asp484, respectively, and Thr13 near the catalytic site. The force constant started at zero and was incremented by 0.1 kcal mol^{-1} Å^{-2} every two ns until the heavy-atom RMSD was less than 1 Å; in the process, the protein and target atoms were kept fixed. Additional structural adjustments were observed during the free 30-ns dynamics.

Comparative analysis of WT and mutants MD simulations

The apo-GalNAc-T1^{D444A}, apo-GalNAc-T1^{D484A}, and apo-GalNAc-T1$^{D444A/D484A}$ systems were created from the apo-GalNAc-T1^{WT} by replacing the corresponding residues in the equilibrated WT conformation. Six metrics were used to assess the changes elicited by the mutations: local side-chain flexibility, hydrophobic/nonpolar interaction networks, H-bond/salt-bridge interaction networks, local backbone conformational changes, and long-range (Pearson's and distance) cross-correlation of side-chain and backbone C_α motions. These quantities are sensitive to thermodynamic conditions and mutations, thus suitable for detecting subtle structural and dynamic changes in comparative analysis. All the calculations were performed with the CHARMM program using the last 20 ns of productive simulation. The values of these metrics were projected on the fourth parameter (B-factor) of the corresponding representative coordinates (PDB format) and visualized as heatmaps in ChimeraX *(66)*. Scripts, structures and ChimeraX sessions are available in Data S2.

LC-MS/MS with HCD-pd-EThcD

Glycopeptides and peptides (0.1 μg) were dissolved in 0.1% TFA and analyzed using an orbitrap Fusion Lumos mass spectrometer connected with Dionex UltiMate 3000 Nano HPLC System (Thermo Fisher Scientific). The nano HPLC system was equipped with an Acclaim™ PepMap™ C18 Nano trap column (3 µm, 100 Å, 75 µm × 2 cm) and separated with an Acclaim™ PepMap™ C18 Nano column (3 µm, 100 Å, 75 µm × 25 cm). The solvent A was 0.1% formic acid in HPLC water, and B was 80% acetonitrile/0.1% formic acid. The LC-MS method duration was 90 min. The LC flow rate was 0.25 µL/min with a linear gradient of 4–40% solvent B over 6-72 min followed by 40-95% B 72-77 min, wash in 95% B 77-82 min, and finally, equilibrated at 1% B for 82-90 min. The spray voltage was positive ion at 1800 V with an ion transfer tube temperature of 250°C. Internal mass calibration was EASY-IC. Advanced peak determination was true. Orbitrap at a resolution of 120K was used to detect precursor masses with a scan range of 250-1800 m/z. The duty cycle was 2 s. Maximum injection time was 50 ms with an AGC target of 400K. RF lens was 30%. Charge states of 2-8 were selected for HCD fragmentation with

a collision energy of 30%, a resolution of 30K, a maximum injection time of 60 ms, an AGC target of 50K, an isolation window of 2, first mass at 110 m/z, and dynamic exclusion of 60 s. If HCD fragmentation generated oxonium ions at 126.055, 138.0549, 144.0655, 168.0654, 186.076, 204.0865, 274.0921, 292.1027, and 366.1395 m/z were detected in the top 20 product ions within 15 ppm, EThcD fragmentation was triggered and acquired in the orbitrap with a collision energy of 30%, a resolution of 30K, a maximum injection time of 200 ms, an AGC target of 100K, a SA collision energy of 35%, ETD reagent target 500K, max ETD reagent injection time of 200 ms, and first mass at 110 m/z. ETD reaction time was 125 ms for charge 2, 100 ms for charge 3, and 75 ms for ≥ charge 4.

LC-MS/MS data analysis for pinpointing glycosylation site

Software packages pGlyco3 *(46)* (release date 2021-06-15) were used to identify glycopeptides. The Muc1 peptide sequence was used for database search. Variable modifications were oxidation (M), HexNAc (S), and HexNAc (T). Carbamidomethylation (C) was the static modification. No enzyme digestion was used in the search. The HCD + ETHCD search mode was selected. The glycopeptide FDR was 0.01. The output data from pGlyco3 were filtered to keep peptide-spectrum matches (PSMs) having HexNAc in the Glycan Composition column. PSMs with the highest EThcD O-glycosite mapping score of at least 0.75 on any serine or threonine residues of peptide sequences were kept. The M/Z and intensity of the peaks in PSMs identified by the pGlyco3 were then exported and filtered to have an intensity greater than 5000. Using these filtered values, they were then imported into the interactive peptide spectra annotator (https://www.interactivepeptidespectralannotator.com/PeptideAnnotator.html) for manual confirmation of peaks corresponding to b and y as well as c and z ions.

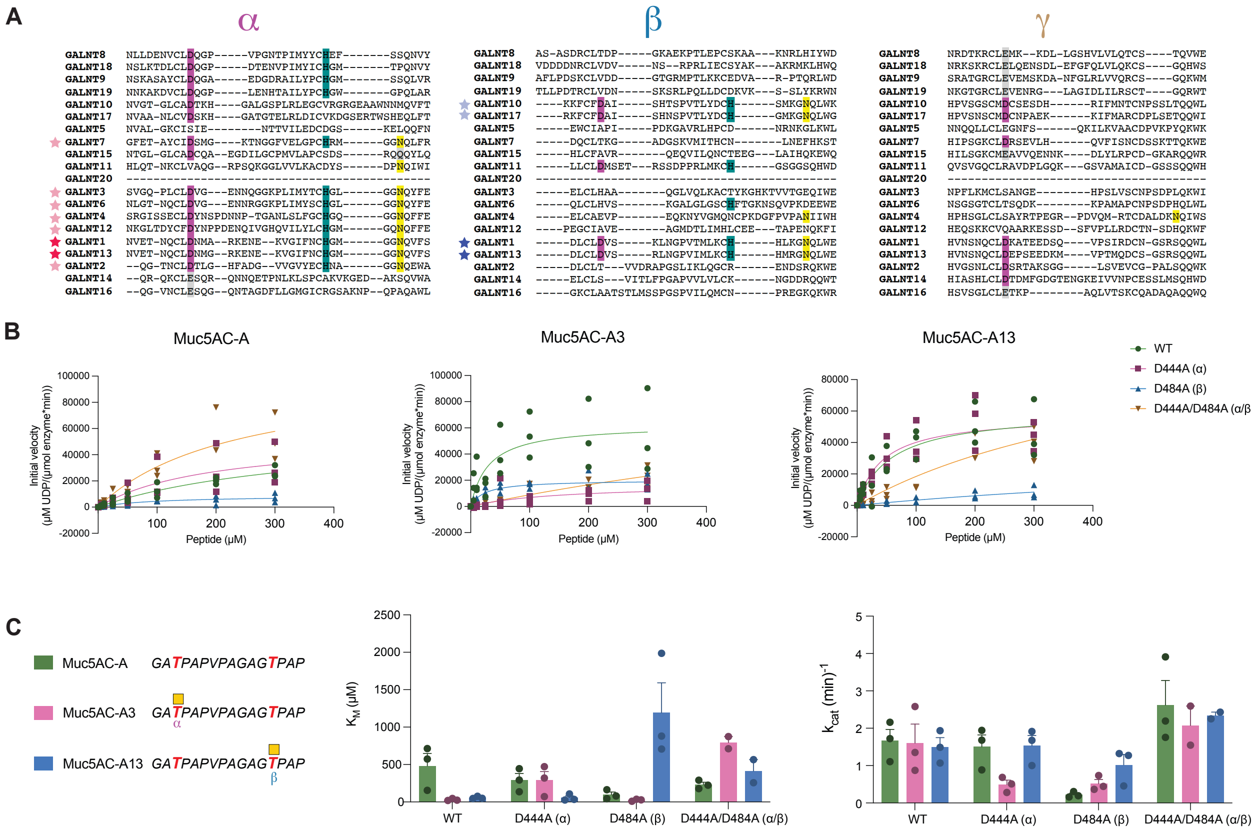

Fig. S1.
(A) Sequence alignment of human GalNAc-T lectin domain repeats. Canonical sugar binding residues (Asp, His, and Asn) are highlighted when present. **(B, C)** Enzyme kinetics of GalNAc-T1^{WT}, GalNAc-T1^{D444A}, GalNAc-T1^{D484A}, and GalNAc-T1$^{D444A/D484A}$ against Muc5AC-A peptides, with the acceptor Thr in red and GalNAc shown in a yellow square.

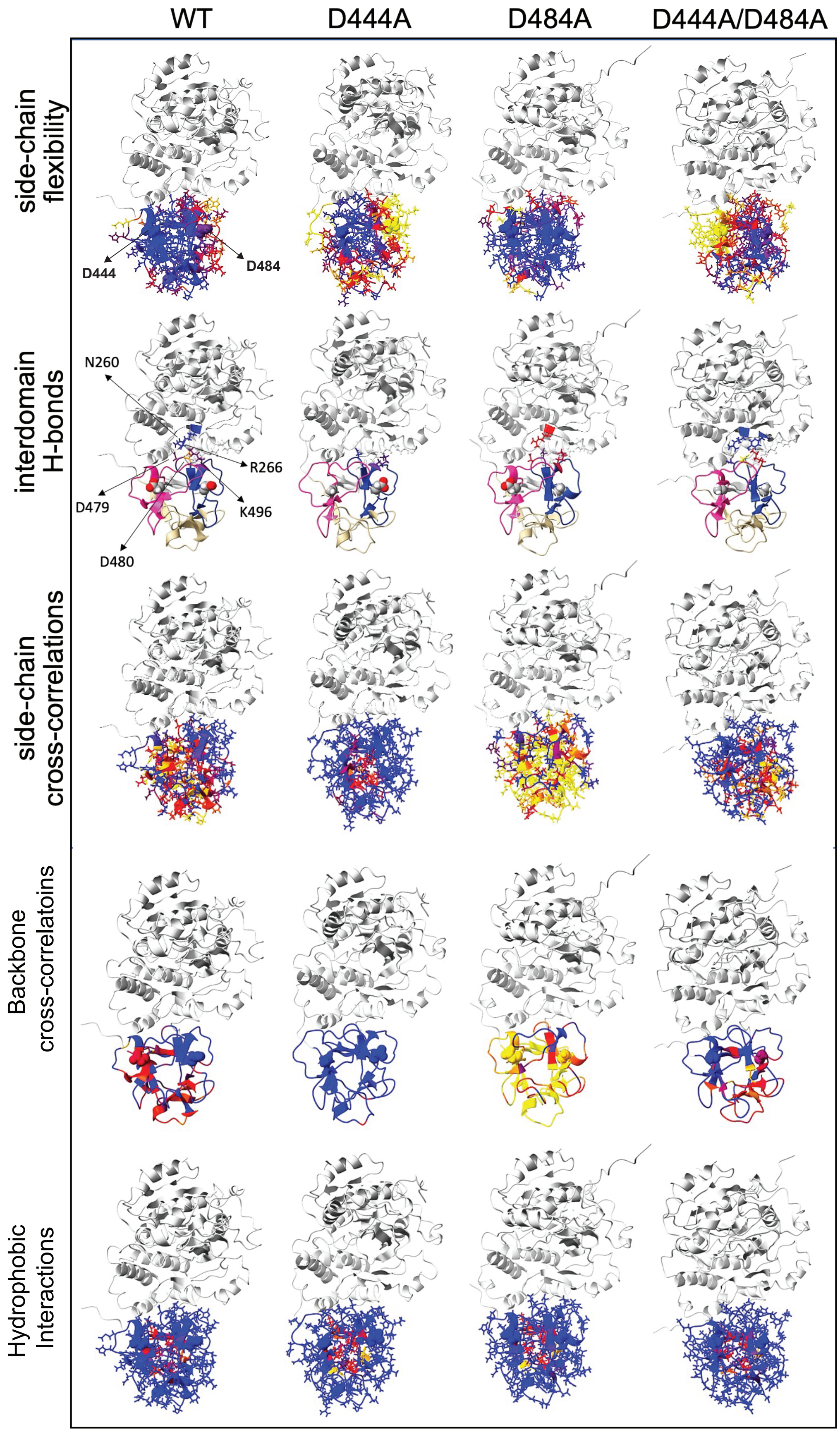

WT
D444A
D484A
D444A/D484A
side-chain flexibility
D444
D484
interdomain H-bonds
N260
R266
D479
K496
D480
side-chain cross-correlations
Backbone cross-correlatoins
Hydrophobic Interactions

Fig. S2.

Full set of metrics used to assess the effects of single and double mutations in the α and β repeats of the catalytic domain. Besides the sidechain flexibility (see main text and Fig. 1D), the other metrics showing noteworthy changes were interfacial lectin/catalytic domains hydrogen bonds (Hb; second row) and and long-range (> 12 Å) cross-correlations of movements (cc; third and four rows). The Hb panel shows the residues that engage/disengage in interdomain interactions, mainly through the β repeat (blue: < 20 %, yellow: > 80 %); two residues in the linker (Asp373 and Arg378; shown white) remains H-bonded to two residues in the catalytic domain (Arg225 and Glu368; white) regardless of the mutation, with the stronger interactions seen in GalNAc-T1^{WT}. Backbone ribbon of the α, β, and γ repeats colored as in Fig. 2A. The cc panels show residues with the lowest (blue) and highest (yellow) long-range cross-correlations with other residues; the complete cross-correlation matrices are added to Data S2. Intra-T1 nonpolar/hydrophobic interactions (bottom row) showed no significant differences. Backbone conformational changes were minor (not shown, see PDB coordinates in the repository), except for the D484A mutant, which displays a mild reconfiguration of the catalytic domain relative to the lectin domain, probably driven by the strengthening of the interdomain H-bond network (see main text).

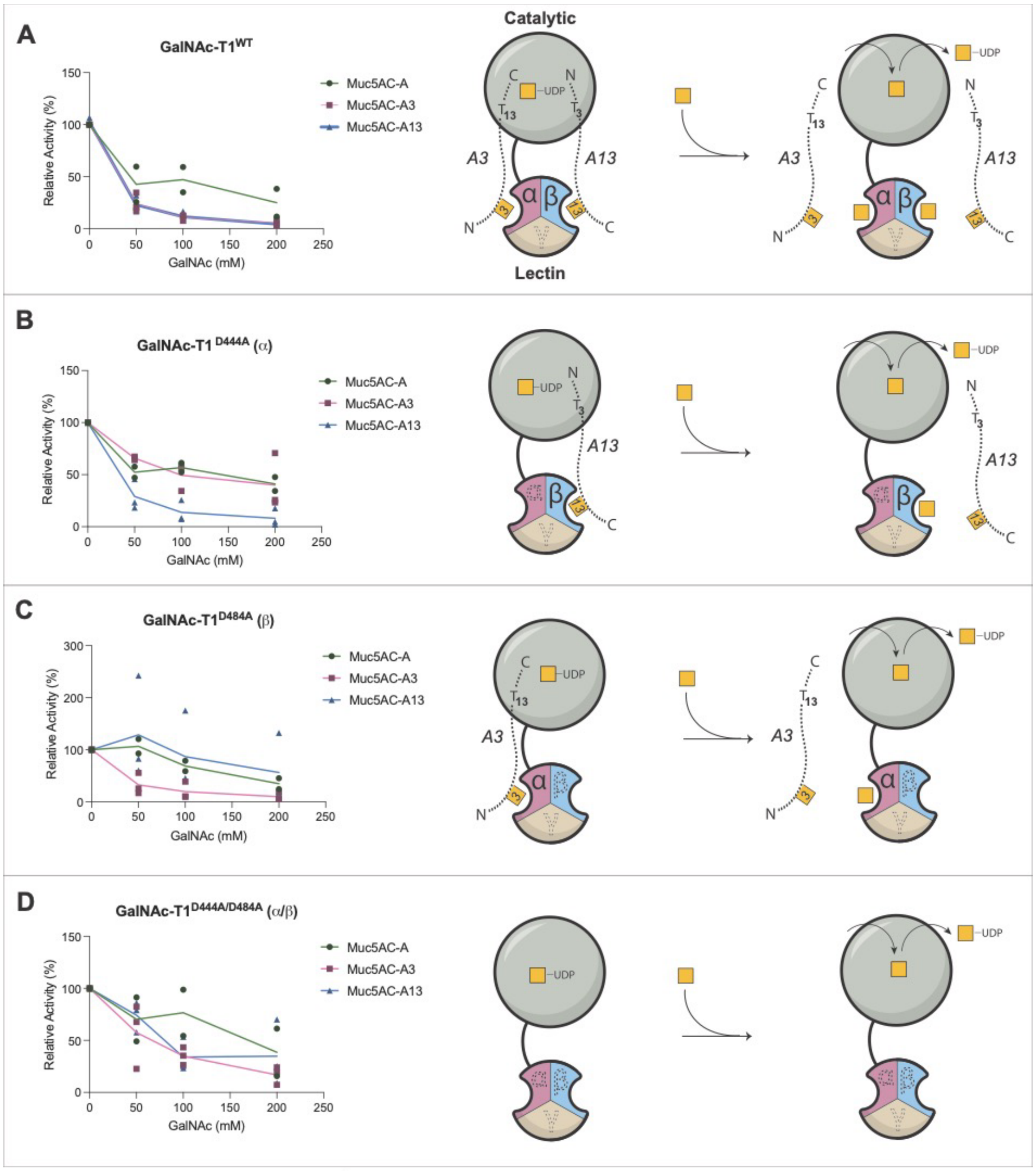

Fig. S3.

(A) GalNAc inhibition assays showing that increasing concentrations of free GalNAc compete with and inhibit (glyco)peptide substrate binding to the lectin domain of GalNAc-T1^{WT}, however, **(B)** we observe a smaller decrease in relative activity for GalNAc-T1^{D444A} and Muc5AC-A3 as well as **(C)** GalNAc-T1^{D484A} and Muc5AC-A13. **(D)** For GalNAc-T1$^{D444A/D484A}$, relative activity is increased for both glycopeptides compared to GalNAc-T1^{WT}. Assays were performed in triplicate and replicated two (Muc5AC-A) or three times (Muc5AC-A3, Muc5AC-A13).

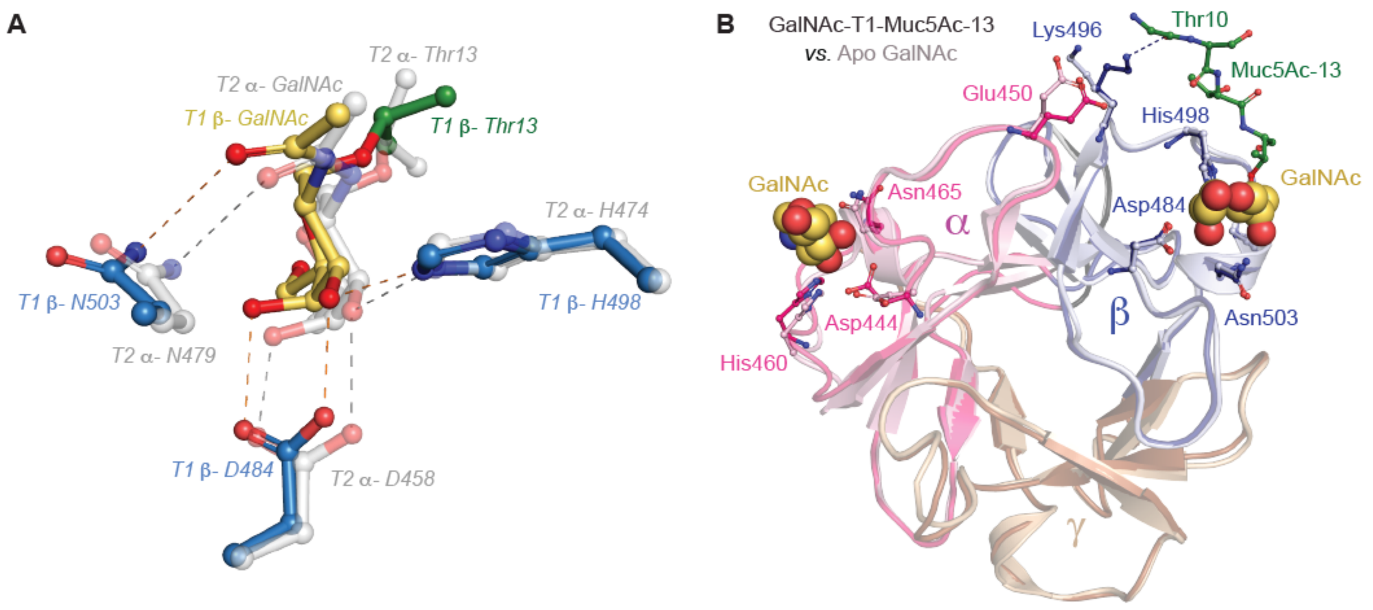

Fig. S4.

(A) Superposition of the β repeat of GalNAc-T1 (dark colors) and the α repeat of GalNAc-T2 (light colors) showing that GalNAc binding residues adopt a similar conformation to interact with Thr-O-GalNAc. **(B)** The lectin repeats of Apo-GalNAc-T1, and substrate bound GalNAc-T1 are superposable, suggesting that Muc5AC-13 glycopeptide binding does not greatly alter the overall lectin domain conformation.

Raw file: 2023_27_1_abbie_1_WT
Experiment condition: naked MUC-1 peptide treated with wild-type GalNAc-T1
Peptide sequence: RPAPGSTAPPAHGVTSAPDTRPAPGSTAPPAHGV
Glycan composition: N(1)
Site localization score: 0.90
Glycosylation site: T15

MS2 HCD scan #5170

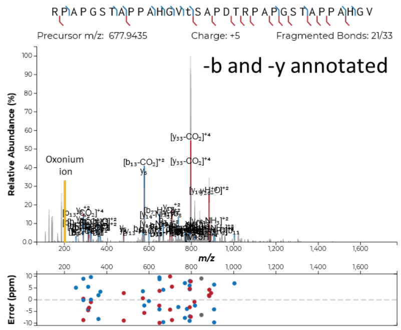

MS2 EThcD scan #5173

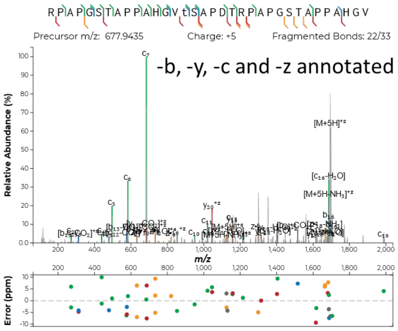

MS2 EThcD scan #5173

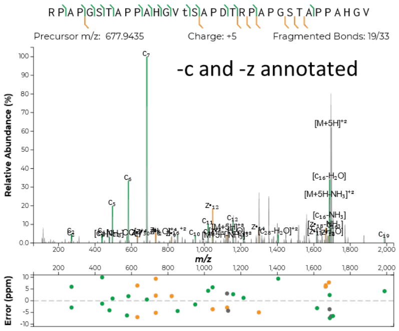

Raw file: 2023_27_1_abbie_1_WT
Experiment condition: naked MUC-1 peptide treated with wild-type GalNAc-T1
Peptide sequence: RPAPGSTAPPAHGVTSAPDTRPAPGSTAPPAHGV
Glycan composition: N(1)
Site localization score: 0.89
Glycosylation site: T15

MS2 HCD scan #5186

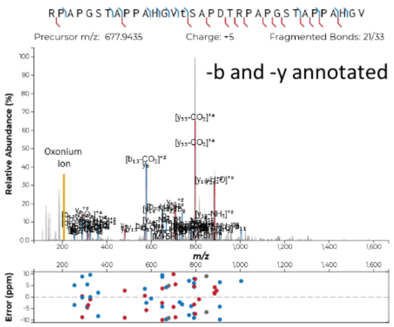

MS2 EThcD scan #5189

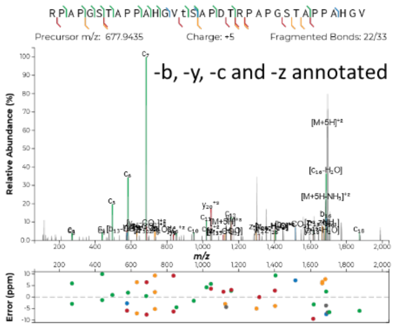

MS2 EThcD scan #5189

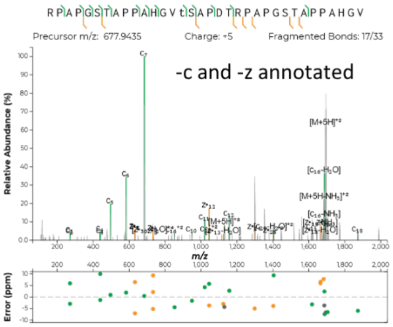

Raw file: 2023_27_1_abbie_1_A
Experiment condition: naked MUC-1 peptide treated with alpha subunit knockout GalNAc-T1
Peptide sequence: RPAPGSTAPPAHGVTSAPDTRPAPGSTAPPAHGV
Glycan composition: N(1)
Site localization score: 0.88
Glycosylation site: T15

MS2 HCD scan #5275

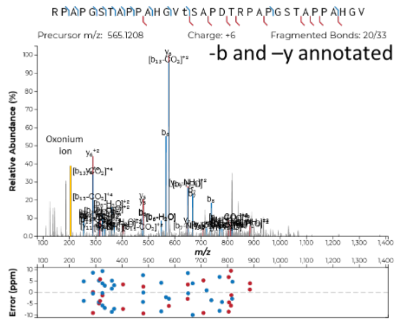

MS2 EThcD scan #5278

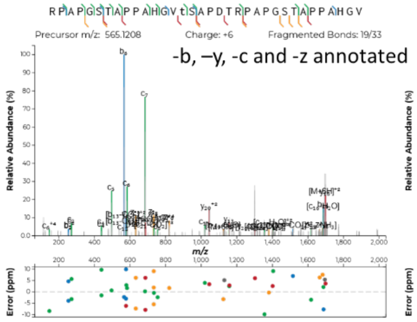

MS2 EThcD scan #5278

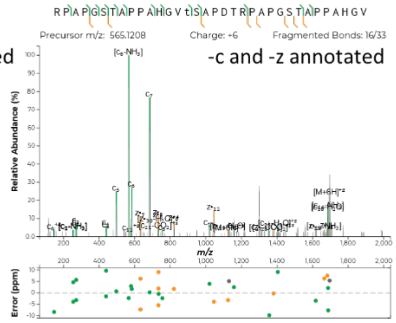

Raw file name: 2023_27_1_abbie_1_A
Experiment condition: naked MUC-1 peptide treated with alpha subunit knockout GalNAc-T1
Peptide sequence: RPAPGSTAPPAHGVTSAPDTRPAPGSTAPPAHGV
Glycan composition: N(1)
Site localization score: 0.86
Glycosylation site: T15

MS2 HCD scan #5234

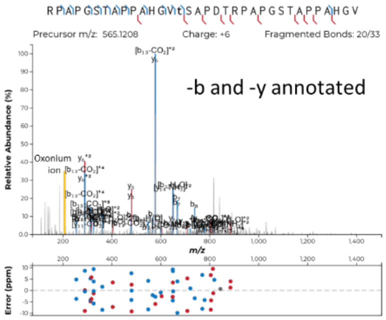

MS2 EThcD scan #5237

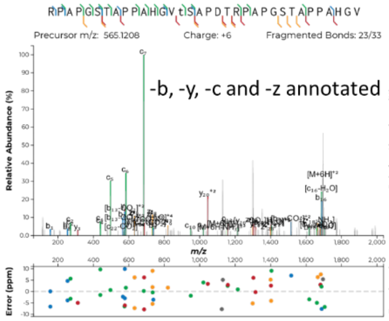

MS2 EThcD scan #5237

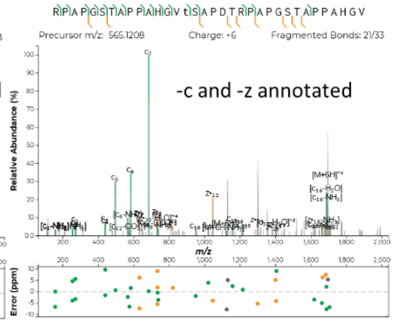

Raw file name: 2023_27_1_abbie_1_B
Experiment condition: naked MUC-1 peptide treated with beta subunit knockout GalNAc-T1
Peptide sequence: RPAPGSTAPPAHGVTSAPDTRPAPGSTAPPAHGV
Glycan composition: N(1)
Site localization score: 0.92
Glycosylation site: T15

MS2 HCD scan #5225

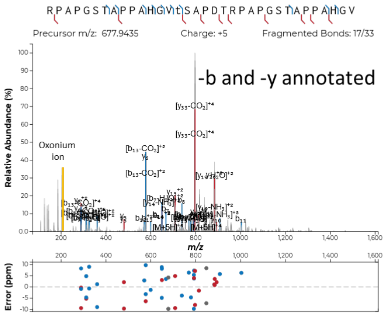

MS2 EThcD scan #5227

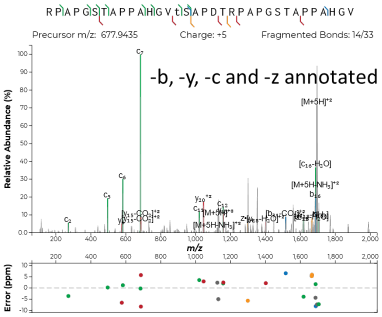

MS2 EThcD scan #5227

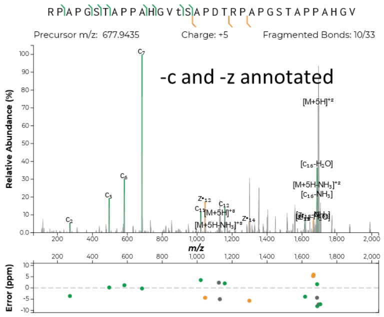

Raw file: 2023_27_1_abbie_1_B
Experiment condition: naked MUC-1 peptide treated with beta subunit knockout GalNAc-T1
Peptide sequence: RPAPGSTAPPAHGVTSAPDTRPAPGSTAPPAHGV
Glycan composition: N(1)
Site localization score: 0.89
Glycosylation site: T15

MS2 HCD scan #5255

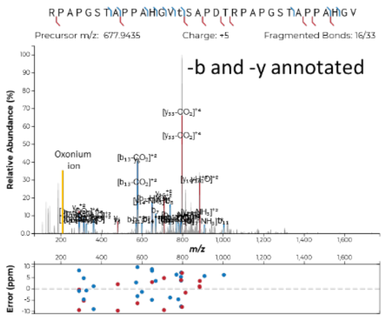

MS2 EThcD scan #5258

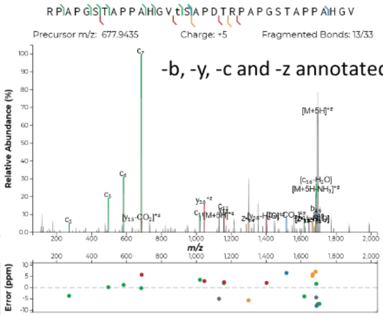

MS2 EThcD scan #5258

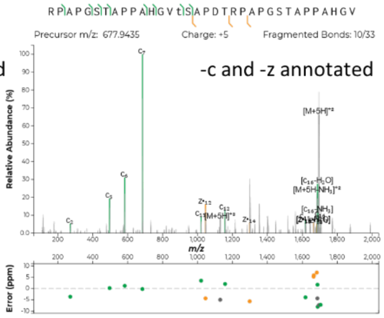

Raw file: 2023_27_1_abbie_1_AB
Experiment condition: naked MUC-1 peptide treated with alpha and beta subunit knockout GalNAc-T1
Peptide sequence: RPAPGSTAPPAHGVTSAPDTRPAPGSTAPPAHGV
Glycan composition: N(1)
Site localization score: 0.92
Glycosylation site: T15

MS2 HCD scan #5017 | MS2 EThcD scan #5019 | MS2 EThcD scan #5019

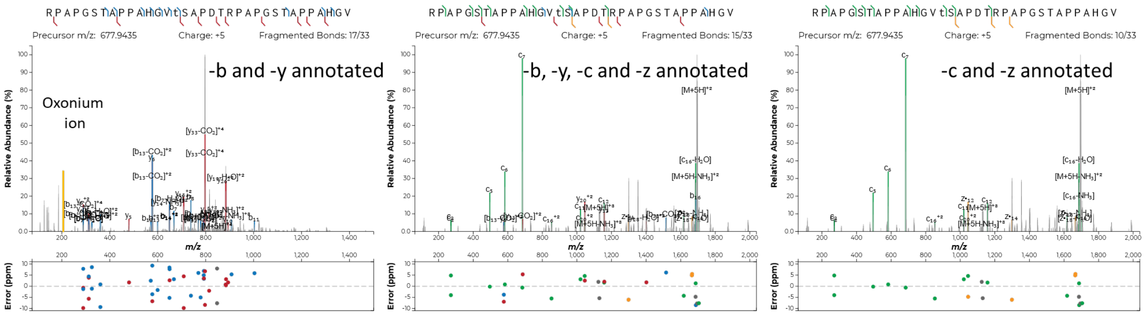

Raw file: 2023_27_1_abbie_1_AB
Experiment condition: naked MUC-1 peptide treated with alpha and beta subunit knockout GalNAc-T1
Peptide sequence: RPAPGSTAPPAHGVTSAPDTRPAPGSTAPPAHGV
Glycan composition: N(1)
Site localization score: 0.85
Glycosylation site: T15

MS2 HCD scan #5083 | MS2 EThcD scan #5086 | MS2 EThcD scan #5086

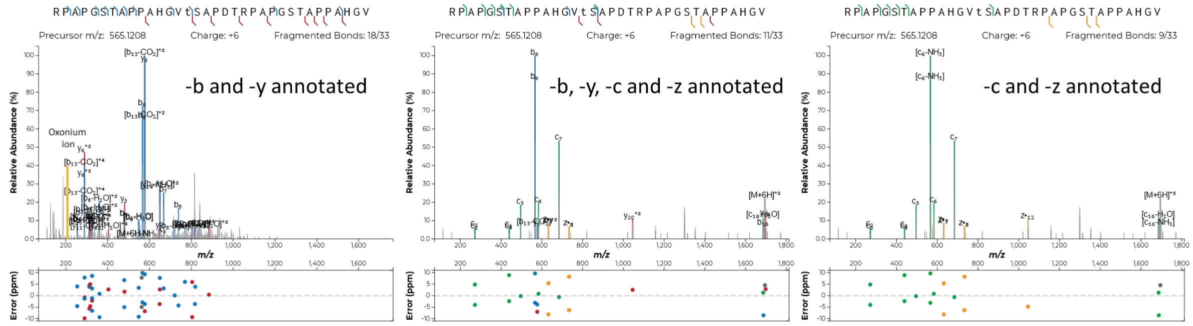

Fig. S5.
Mass spectrometry data showing the identification of Thr15 as the only glycosylation site on Muc1 peptide after treatment with WT or variants of GalNAc-T1.

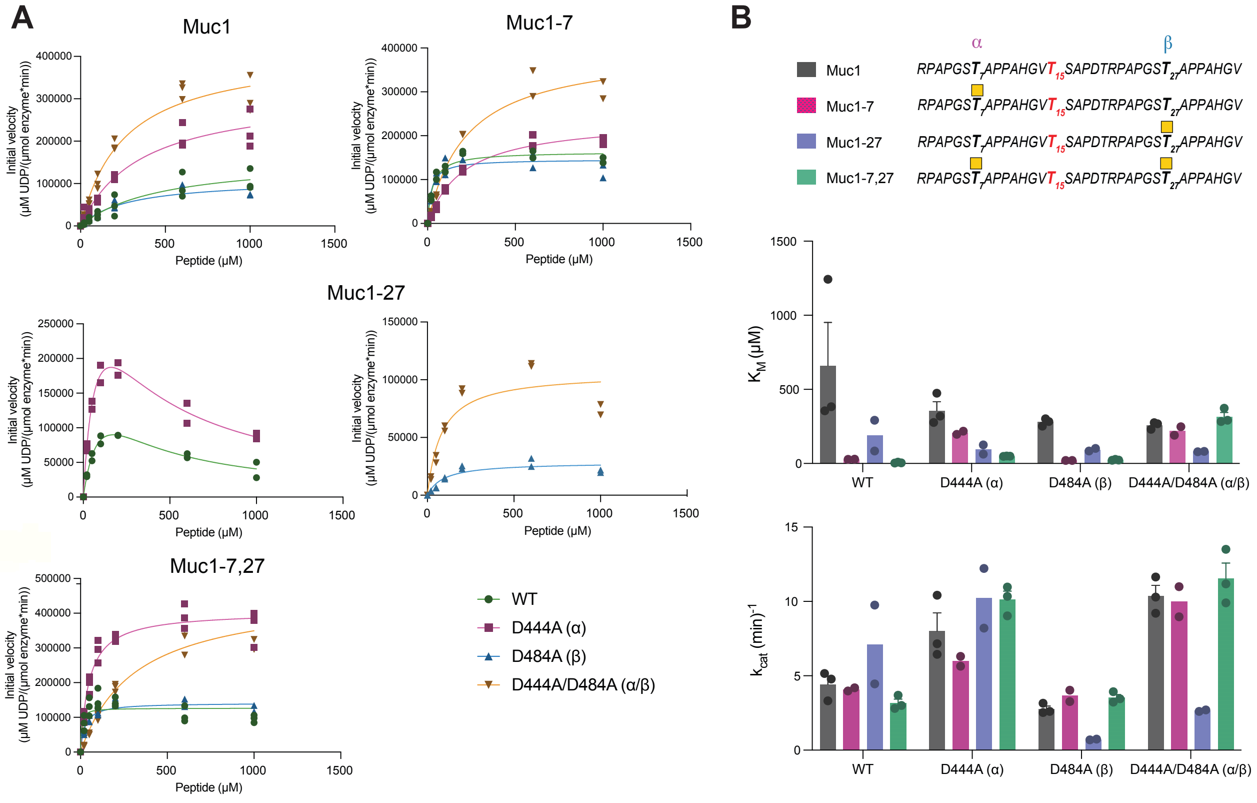

Fig. S6.

(A, B) Enzyme kinetics of GalNAc-T1^{WT}, GalNAc-T1^{D444A}, GalNAc-T1^{D484A}, and GalNAc-T1$^{D444A/D484A}$ against Muc1 peptides displaying lectin domain mediated bidirectionality and a synergistic enhancement from the α and β repeats in di-glycosylated substrate Muc1-7,27 as described in Fig. 3A. Acceptor Thr is colored red, GalNAc is depicted as yellow squares.

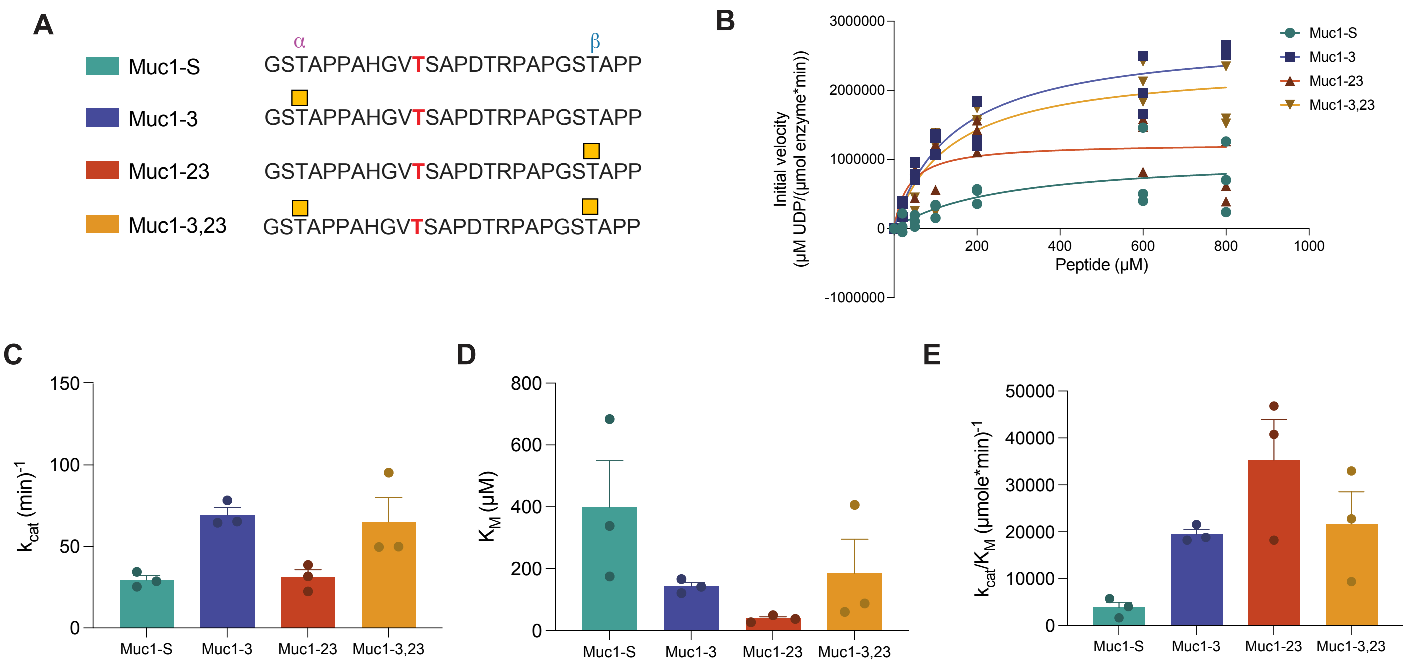

Fig. S7.

Enzyme kinetics of GalNAc-T2 using a series of Muc1 peptides and glycopeptides as substrates, with the putative acceptor Thr shown in red and GalNAc as a yellow square **(A-D)**. **(E)** GalNAc-T2, which contains a single active GalNAc binding lectin repeat (α), has a similar k_{cat}/K_M with Muc1 mono- and di-glycopeptides. GalNAc-T2 assays were performed in duplicate and replicated three times.

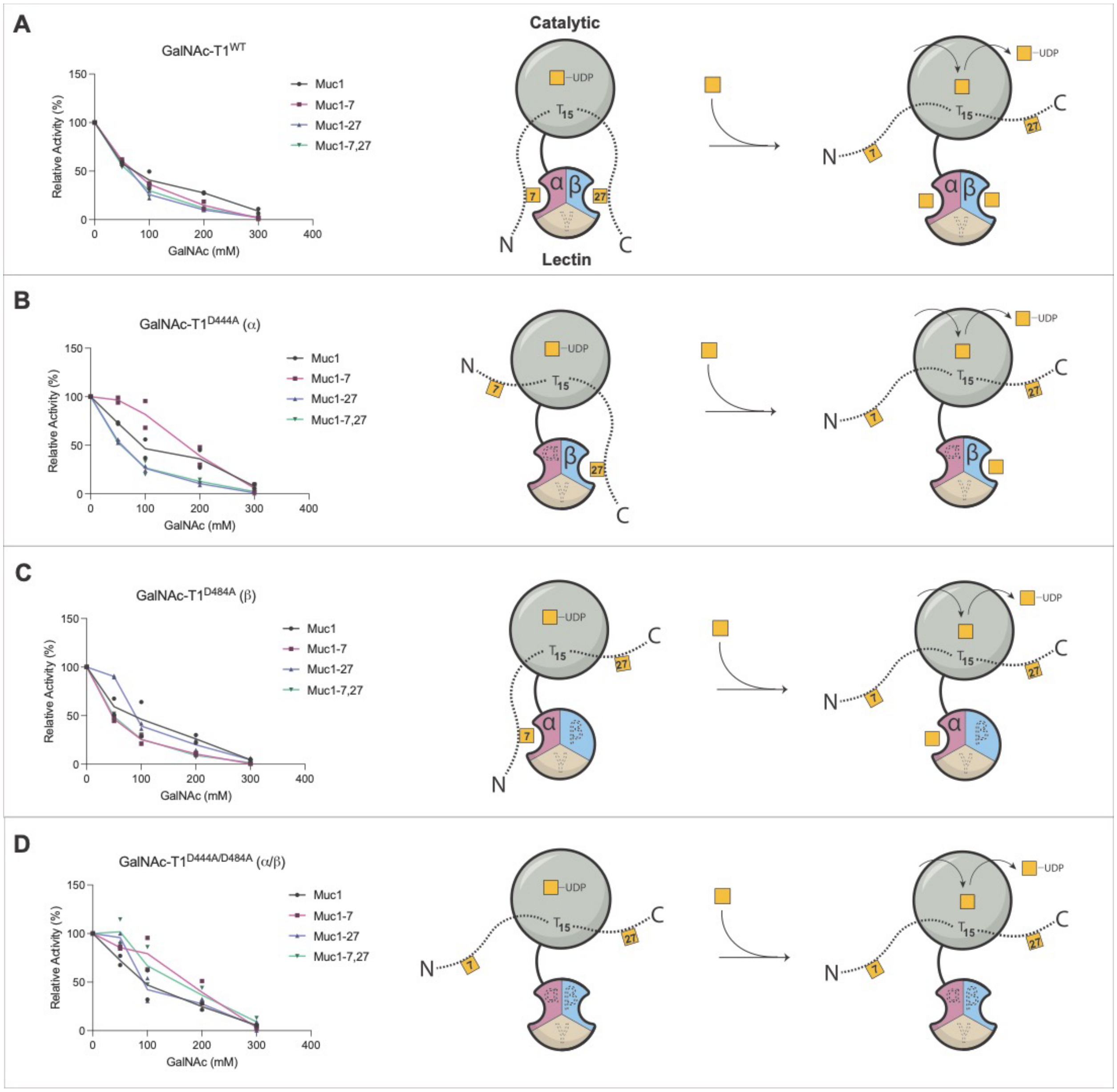

Fig. S8.
GalNAc inhibition assay showing disrupted GalNAc binding between WT and variants of GalNAc-T1 to Muc1 peptides and glycopeptides upon mutation of the α and/or β repeats **(A-D)**. Assays were performed in duplicate and replicated two times.

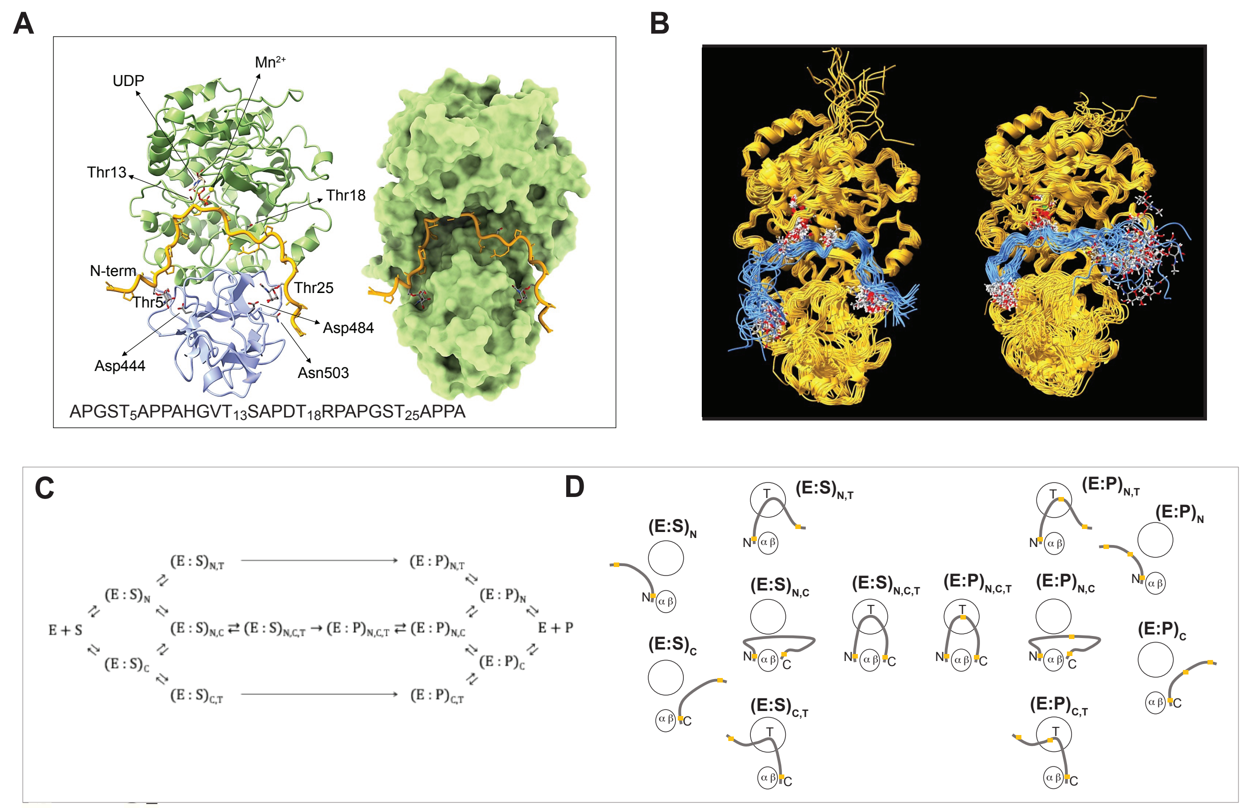

Fig. S9.

(A) Snapshot at the end of the dynamics simulation showing the di-glycosylated Muc1 with the two GalNAc bound to their respective pockets in the α and β repeats of WT GalNAc-T1 (left: ribbon representation; catalytic domain colored green; lectin domain, blue. Right: the same snapshot showing the molecular surface of GalNAc-T1, with the deep peptide-wrapping crevice at the lectin/catalytic domain boundary that further stabilizes the complex). **(B)** Snapshots at equal time intervals along the 30-ns trajectories of two simulations, one in which the two GalNAc remains bound to their respective sites and one in which the C-term GalNAc becomes detached**.** **(C)** Proposed kinetic model based on the simulations and experimental results; all the proposed paths are likely to co-exist, with mutations modulating each; expression for k_{cat} and K_M can be formally derived from the corresponding kinetic equations after suitable approximations (e.g., MM assumptions); although other steps known to be involved, e.g., opening/closing of activation loop and detachment of UDP, are implicit in the diagram but can be explicitly factored in. **(D)** Definitions of each of the steps depicted in **(C).**

Table S1.

Enzyme kinetics for the Muc5AC-A peptides and GalNAc-T1 variants

		WT	D444A (α)	D484A (β)	D444A/D484A (α/β)
Muc5Ac-A	*Vmax (μM/(μmole*min))*	66526 ± 12231	60156 ± 12696	8835 ± 1765	104795 ± 26306
	K_M *(μM)*	481 ± 168	292 ± 89	97 ± 35	231 ± 33
	k_{cat} *(min^{-1})*	1.7 ± 0.3	1.5 ± 0.3	0.2 ± 0.04	2.6 ± 0.7
	k_{cat}/K_M *((μmole*min)$^{-1}$)*	176 ± 55	280 ± 144	109 ± 32	441 ± 49
Muc5Ac-A3	*Vmax (μM/(μmole*min))*	64106 ± 20301	19695 ± 4683	20618 ± 4503	82845 ± ND
	K_M *(μM)*	30 ± 8	288 ± 117	25 ± 8	793 ± ND
	k_{cat} *(min^{-1})*	1.6 ± 0.5	0.5 ± 0.1	0.5 ± 0.1	2.1 ± ND
	k_{cat}/K_M *((μmole*min)$^{-1}$)*	2077 ± 135	133 ± 80	1010 ± 307	108 ± ND
Muc5Ac-A13	*Vmax (μM/(μmole*min))*	59901 ± 10043	61262 ± 10915	40722 ± 11253	93621 ± ND
	K_M *(μM)*	58 ± 9	56 ± 26	1191 ± 400	411 ± ND
	k_{cat} *(min^{-1})*	1.5 ± 0.3	1.5 ± 0.3	1.0 ± 0.3	2.3 ± ND
	k_{cat}/K_M *((μmole*min)$^{-1}$)*	1078 ± 240	1372 ± 352	37 ± 11	268 ± ND

Table S2.
Crystal Diffraction and Refinement Data

	GalNAc-T1-Muc5AC-13-Mn^{2+}- UDP PDB ID 8V9Q
**Data collection**	
Space group	P21
Cell dimensions	
a, b, c (Å)	59.6, 72.8, 148.5
a, b, γ (°)	90, 95.4, 90
Resolution (Å) [1]	29.68 - 2.29 (2.34-2.29)
R_{pim} [1]	0.068 (0.545)
$I/\sigma I$ [1]	10.0 (1.07)
$CC^{1/2}$ [1]	0.982 (0.410)
Completeness (%) [1]	97.4 (86.0)
Redundancy [1]	3.5 (2.7)
No. unique reflections [1]	55051 (2410)
**Refinement**	
R_{work} / R_{free}	24.8/27.9
No. atoms	
Protein	15147
Mn^{2+}	2
UDP	72
Peptide / 2-acetamido-2-deoxy-β-D-glucopyranose	479/83
2-acetamido-2-deoxy-β-D-glucopyranose	163
β-D-mannopyranose	42
α-D-mannopyranose	19
Water / Solvent	120/77
B-factors	
Average	79.6
Macromolecules	80.3
Solvent[2]	39.9
Ligands	75.8
R.m.s deviations	
Bond lengths (Å)	0.004
Bond angles (°)	0.801

[1] Data in the highest resolution shell is shown in the parenthesis.

[2] Water, ethylene glycol and glycerol.

Table S3.

Enzyme Kinetics for the Muc1 Peptides and GalNAc-T1

		WT	D444A (α)	D484A (β)	D444A/D484A (α/β)
Muc1	*Vmax (µM/(µmole*min))*	176750 ± 22442	320078 ± 48958	110428 ± 7986	415208 ± 28120
	K_M *(µM)*	661 ± 291	357 ± 60	279 ± 15	258 ± 14
	k_{cat} (min^{-1})	4.4 ± 0.56	8.0 ± 1.2	2.8 ± 0.2	10.4 ± 0.7
	k_{cat}/K_M $((\mu mole*min)^{-1})$	350 ± 107	901 ± 15	401 ± 51	1610 ± 70
Muc1-7	*Vmax (µM/(µmole*min))*	163390 ± 4206	238929 ± 13325	146003 ± 15097	399297 ± 40322
	K_M *(µM)*	29 ± 0.5	207 ± 11	21 ± 0.4	219 ± 29
	k_{cat} (min^{-1})	4.1 ± 0.11	6.0 ± 0.34	3.7 ± 0.38	10 ± 1
	k_{cat}/K_M $((\mu mole*min)^{-1})$	5733 ± 238	1154 ± 8	7050 ± 855	1828 ± 59
Muc1-27	*Vmax (µM/(µmole*min))*	284494 ± 105915	408677 ± 80173	28517 ± 982	106062 ± 2183
	K_M *(µM)*	188 ± 104	95 ± 31	93 ± 9	81 ± 1
	k_{cat} (min^{-1})	7.1 ± 2.7	10.2 ± 2	0.72 ± 0.03	2.7 ± 0.06
	k_{cat}/K_M $((\mu mole*min)^{-1})$	1733 ± 396	4534 ± 645	310 ± 19	1311 ± 44
Muc1-7,27	*Vmax (µM/(µmole*min))*	126653 ± 10684	404727 ± 22853	141626 ± 8272	461332 ± 42190
	K_M *(µM)*	5 ± 2	50 ± 0.7	24 ± 2	316 ± 29
	k_{cat} (min^{-1})	3.2 ± 0.27	10.1 ± 0.57	3.5 ± 0.21	11.5 ± 1.1
	k_{cat}/K_M $((\mu mole*min)^{-1})$	28334 ± 8370	8176 ± 521	5859 ± 518	1463 ± 64

Table S4.
Enzyme Kinetics for Muc1 Peptides and GalNAc-T2

		GalNAc-T2
Muc1-S	*Vmax (µM/(µmole*min))*	1177979 ± 106652
	K_M *(µM)*	399 ± 150
	k_{cat} *(min^{-1})*	29 ± 2.7
	k_{cat}/K_M *(($µmole*min)^{-1}$)*	3842 ± 1190
Muc1-3	*Vmax (µM/(µmole*min))*	2774605 ± 178590
	K_M *(µM)*	143 ± 13
	k_{cat} *(min^{-1})*	69 ± 4.5
	k_{cat}/K_M *(($µmole*min)^{-1}$)*	19527 ± 1022
Muc1-23	*Vmax (µM/(µmole*min))*	1237882 ± 187774
	K_M *(µM)*	38 ± 6
	k_{cat} *(min^{-1})*	31 ± 4.7
	k_{cat}/K_M *(($µmole*min)^{-1}$)*	35278 ± 8709
Muc1-3,23	*Vmax (µM/(µmole*min))*	2600887 ± 606336
	K_M *(µM)*	185 ± 111
	k_{cat} *(min^{-1})*	65 ± 15
	k_{cat}/K_M *(($µmole*min)^{-1}$)*	21704 ± 6831

Table S5.
Primers for human GalNAc-T1 Mutant Constructs

Construct	5'-Primer	3'-Primer
His_6-TEV-GalNAc-T1^{D444A}	CAGTGTCTAGCTAACATG GCTAGAAAAGAGAATG	CTAGCCATGTTAGCTAG ACACTGATTCGTTTCCA CATTTCG
His_6-TEV-GalNAc-T1^{D484A}	CCTTTGCTTGGCTGTTTCC AAACTTAATGGCCCAG	GTTTGGAAACAGCCAA GCAAAGGTCATCTGTTC
His_6-TEV-GalNAc-T1$^{D444A/D484A}$	CCTTTGCTTGGCTGTTTCC AAACTTAATGGCCCAG	GTTTGGAAACAGCCAA GCAAAGGTCATCTGTTC

Data S1. (Separate folder): Kinetics raw data and biostatistics calculations

Data S2. (Separate folder): Modeling and simulations

Data S3. (Separate file): Mass spectrometry data

REFERENCES AND NOTES

1. H. H. Wandall, M. A. I. Nielsen, S. King-Smith, N. de Haan, I. Bagdonaite, Global functions of O-glycosylation: Promises and challenges in O-glycobiology. *FEBS J.* **288**, 7183–7212 (2021).

2. M. R. Kudelka, T. Ju, J. Heimburg-Molinaro, R. D. Cummings, Simple sugars to complex disease—Mucin-type O-glycans in cancer. *Adv. Cancer Res.* **126**, 53–135 (2015).

3. M. R. M. Hussain, D. C. Hoessli, M. Fang, N-acetylgalactosaminyltransferases in cancer. *Oncotarget* **7**, (2016), 54067, 54081.

4. K. Kato, L. Hansen, H. Clausen, Polypeptide N-acetylgalactosaminyltransferase-associated phenotypes in mammals. *Molecules* **26**, (2021).

5. Y. Zhang, L. Wang, D. K. W. Ocansey, B. Wang, L. Wang, Z. Xu, Mucin-type O-glycans: Barrier, microbiota, and immune anchors in inflammatory bowel disease. *J. Inflamm. Res.* **Volume 14**, 5939–5953 (2021).

6. T. Sorensen, T. White, H. H. Wandall, A. K. Kristensen, P. Roepstorff, H. Clausen, UDP-N-acetyl-alpha-D-galactosamine:polypeptide N-acetylgalactosaminyltransferase. Identification and separation of two distinct transferase activities. *J. Biol. Chem.* **270**, 24166–24173 (1995).

7. M. de Las Rivas, E. Lira-Navarrete, T. A. Gerken, R. Hurtado-Guerrero, Polypeptide GalNAc-Ts: From redundancy to specificity. *Curr. Opin. Struct. Biol.* **56**, 87–96 (2019).

8. E. P. Bennett, U. Mandel, H. Clausen, T. A. Gerken, T. A. Fritz, L. A. Tabak, Control of mucin-type O-glycosylation: A classification of the polypeptide GalNAc-transferase gene family. *Glycobiology* **22**, 736–756 (2012).

9. J. Raman, Y. Guan, C. L. Perrine, T. A. Gerken, L. A. Tabak, UDP-N-acetyl-α-d-galactosamine:polypeptide N-acetylgalactosaminyltransferases: Completion of the family tree. *Glycobiology* **22**, 768–777 (2012).

10. K. G. Ten Hagen, T. A. Fritz, L. A. Tabak, All in the family: The UDP-GalNAc:polypeptide N-acetylgalactosaminyltransferases. *Glycobiology* **13**, 1R-16 (2002).

11. B. Hazes, The (QxW)3 domain: A flexible lectin scaffold. *Protein Sci.* **5**, 1490–1501 (1996).

12. A. Imberty, V. Piller, F. Piller, C. Breton, Fold recognition and molecular modeling of a lectin-like domain in UDP GalNAc:polypeptide N-acetylgalactosaminyltransferases. *Protein Eng.* **10**, 1353–1356 (1997).

13. H. Hassan, C. A. Reis, E. P. Bennett, E. Mirgorodskaya, P. Roepstorff, M. A. Hollingsworth, J. Burchell, J. Taylor-Papadimitriou, H. Clausen, The lectin domain of UDP-N-acetyl-D-galactosamine: Polypeptide N-acetylgalactosaminyltransferase-T4 directs its glycopeptide specificities. *J. Biol. Chem.* **275**, 38197–38205 (2000).

14. J. Raman, T. A. Fritz, T. A. Gerken, O. Jamison, D. Live, M. Liu, L. A. Tabak, The catalytic and lectin domains of UDP-GalNAc:polypeptide alpha-N-Acetylgalactosaminyltransferase function in concert to direct glycosylation site selection. *J. Biol. Chem.* **283**, 22942–22951 (2008).

15. T. A. Gerken, L. Revoredo, J. J. Thome, L. A. Tabak, M. B. Vester-Christensen, H. Clausen, G. K. Gahlay, D. L. Jarvis, R. W. Johnson, H. A. Moniz, K. Moremen, The lectin domain of the polypeptide GalNAc transferase family of glycosyltransferases (ppGalNAc Ts) acts as a switch directing glycopeptide substrate glycosylation in an N- or C-terminal direction, further controlling mucin type O-glycosylation. *J. Biol. Chem.* **288**, 19900–19914 (2013).

16. L. Revoredo, S. Wang, E. P. Bennett, H. Clausen, K. W. Moremen, D. L. Jarvis, K. G. Ten Hagen, L. A. Tabak, T. A. Gerken, Mucin-type O-glycosylation is controlled by short- and long-range glycopeptide substrate recognition that varies among members of the polypeptide GalNAc transferase family. *Glycobiology* **26**, 360–376 (2016).

17. H. H. Wandall, F. Irazoqui, M. A. Tarp, E. P. Bennett, U. Mandel, H. Takeuchi, K. Kato, T. Irimura, G. Suryanarayanan, M. A. Hollingsworth, H. Clausen, The lectin domains of polypeptide GalNAc-transferases exhibit carbohydrate-binding specificity for GalNAc: Lectin

binding to GalNAc-glycopeptide substrates is required for high density GalNAc-O-glycosylation. *Glycobiology* **17**, 374–387 (2007).

18. J. W. Pedersen, E. P. Bennett, K. T. Schjoldager, M. Meldal, A. P. Holmer, O. Blixt, E. Clo, S. B. Levery, H. Clausen, H. H. Wandall, Lectin domains of polypeptide GalNAc transferases exhibit glycopeptide binding specificity. *J. Biol. Chem.* **286**, 32684–32696 (2011).

19. Y. Kong, H. J. Joshi, K. T. Schjoldager, T. D. Madsen, T. A. Gerken, M. B. Vester-Christensen, H. H. Wandall, E. P. Bennett, S. B. Levery, S. Y. Vakhrushev, H. Clausen, Probing polypeptide GalNAc-transferase isoform substrate specificities by in vitro analysis. *Glycobiology* **25**, 55–65 (2015).

20. T. A. Gerken, O. Jamison, C. L. Perrine, J. C. Collette, H. Moinova, L. Ravi, S. D. Markowitz, W. Shen, H. Patel, L. A. Tabak, Emerging paradigms for the initiation of mucin-type protein O-glycosylation by the polypeptide GalNAc transferase family of glycosyltransferases. *J. Biol. Chem.* **286**, 14493–14507 (2011).

21. T. A. Gerken, J. Raman, T. A. Fritz, O. Jamison, Identification of common and unique peptide substrate preferences for the UDP-GalNAc:polypeptide alpha-N-acetylgalactosaminyltransferases T1 and T2 derived from oriented random peptide substrates. *J. Biol. Chem.* **281**, 32403–32416 (2006).

22. B. C. O'Connell, F. K. Hagen, L. A. Tabak, The influence of flanking sequence on the O-glycosylation of threonine in vitro. *J. Biol. Chem.* **267**, 25010–25018 (1992).

23. H. Block, K. Ley, A. Zarbock, Severe impairment of leukocyte recruitment in ppGalNAcT-1-deficient mice. *J. Immunol.* **188**, 5674–5681 (2012).

24. C. M. Phelan, Y. Y. Tsai, E. L. Goode, R. A. Vierkant, B. L. Fridley, J. Beesley, X. Q. Chen, P. M. Webb, S. Chanock, D. W. Cramer, K. Moysich, R. P. Edwards, J. Chang-Claude, M. Garcia-Closas, H. Yang, S. Wang-Gohrke, R. Hein, A. C. Green, J. Lissowska, M. E. Carney, G. Lurie, L. R. Wilkens, R. B. Ness, C. L. Pearce, A. H. Wu, D. J. Van Den Berg, D. O. Stram, K. L. Terry, D. C. Whiteman, A. S. Whittemore, R. A. DiCioccio, V. McGuire, J. A. Doherty, M. A.

Rossing, H. Anton-Culver, A. Ziogas, C. Hogdall, E. Hogdall, S. Kruger Kjaer, J. Blaakaer, L. Quaye, S. J. Ramus, I. Jacobs, H. Song, P. D. Pharoah, E. S. Iversen, J. R. Marks, M. C. Pike, S. A. Gayther, J. M. Cunningham, M. T. Goodman, J. M. Schildkraut, G. Chenevix-Trench, A. Berchuck, T. A. Sellers, A. C. S. Ovarian Cancer Association Consortium, G. Australian Ovarian Cancer Study, Polymorphism in the GALNT1 gene and epithelial ovarian cancer in non-Hispanic white women: The ovarian cancer association consortium. *Cancer Epidemiol. Biomarkers Prev.* **19**, 600–604 (2010).

25. T. A. Sellers, Y. Huang, J. Cunningham, E. L. Goode, R. Sutphen, R. A. Vierkant, L. E. Kelemen, Z. S. Fredericksen, M. Liebow, V. S. Pankratz, L. C. Hartmann, J. Myer, E. S. Iversen, Jr., J. M. Schildkraut, C. Phelan, Association of single nucleotide polymorphisms in glycosylation genes with risk of epithelial ovarian cancer. *Cancer Epidemiol. Biomarkers Prev.* **17**, 397–404 (2008).

26. E. J. Simon, A. D. Linstedt, Site-specific glycosylation of Ebola virus glycoprotein by human polypeptide GalNAc-transferase 1 induces cell adhesion defects. *J. Biol. Chem.* **293**, 19866–19873 (2018).

27. M. Tenno, K. Ohtsubo, F. K. Hagen, D. Ditto, A. Zarbock, P. Schaerli, U. H. von Andrian, K. Ley, D. Le, L. A. Tabak, J. D. Marth, Initiation of protein O glycosylation by the polypeptide GalNAcT-1 in vascular biology and humoral immunity. *Mol. Cell. Biol.* **27**, 8783–8796 (2007).

28. E. Tian, M. P. Hoffman, K. G. Ten Hagen, O-glycosylation modulates integrin and FGF signalling by influencing the secretion of basement membrane components. *Nat. Commun.* **3**, 869 (2012).

29. E. Tian, S. R. Stevens, Y. Guan, D. A. Springer, S. A. Anderson, M. F. Starost, V. Patel, K. G. Ten Hagen, L. A. Tabak, Galnt1 is required for normal heart valve development and cardiac function. *PLOS ONE* **10**, e0115861 (2015).

30. L. Zhang, B. Lv, X. Shi, G. Gao, High expression of N-acetylgalactosaminyl-transferase 1 (GALNT1) associated with invasion, metastasis, and proliferation in osteosarcoma. *Med. Sci. Monit.* **26**, e927837 (2020).

31. L. Zhang, M. Mann, Z. A. Syed, H. M. Reynolds, E. Tian, N. L. Samara, D. C. Zeldin, L. A. Tabak, K. G. Ten Hagen, Furin cleavage of the SARS-CoV-2 spike is modulated by O-glycosylation. *Proc. Natl. Acad. Sci. U.S.A.* **118**, (2021).

32. E. Gonzalez-Rodriguez, M. Zol-Hanlon, G. Bineva-Todd, A. Marchesi, M. Skehel, K. E. Mahoney, C. Roustan, A. Borg, L. Di Vagno, S. Kjær, A. G. Wrobel, D. J. Benton, P. Nawrath, S. L. Flitsch, D. Joshi, A. M. González-Ramírez, K. A. Wilkinson, R. J. Wilkinson, E. C. Wall, R. Hurtado-Guerrero, S. A. Malaker, B. Schumann, O-linked sialoglycans modulate the proteolysis of SARS-CoV-2 spike and likely contribute to the mutational trajectory in variants of concern. *ACS Cent. Sci.* **9**, 393–404 (2023).

33. M. F. Festari, F. Trajtenberg, N. Berois, S. Pantano, L. Revoredo, Y. Kong, P. Solari-Saquieres, Y. Narimatsu, T. Freire, S. Bay, C. Robello, J. Benard, T. A. Gerken, H. Clausen, E. Osinaga, Revisiting the human polypeptide GalNAc-T1 and T13 paralogs. *Glycobiology* **27**, 140–153 (2017).

34. M. Tenno, F. J. Kezdy, A. P. Elhammer, A. Kurosaka, Function of the lectin domain of polypeptide N-acetylgalactosaminyltransferase 1. *Biochem. Biophys. Res. Commun.* **298**, 755–759 (2002).

35. M. Tenno, A. Saeki, F. J. Kezdy, A. P. Elhammer, A. Kurosaka, The lectin domain of UDP-GalNAc:polypeptide N-acetylgalactosaminyltransferase 1 is involved in O-glycosylation of a polypeptide with multiple acceptor sites. *J. Biol. Chem.* **277**, 47088–47096 (2002).

36. E. Lira-Navarrete, M. de Las Rivas, I. Companon, M. C. Pallares, Y. Kong, J. Iglesias-Fernandez, G. J. Bernardes, J. M. Peregrina, C. Rovira, P. Bernado, P. Bruscolini, H. Clausen, A. Lostao, F. Corzana, R. Hurtado-Guerrero, Dynamic interplay between catalytic and lectin domains of GalNAc-transferases modulates protein O-glycosylation. *Nat. Commun.* **6**, 6937 (2015).

37. A. J. Fernandez, E. J. P. Daniel, S. P. Mahajan, J. J. Gray, T. A. Gerken, L. A. Tabak, N. L. Samara, The structure of the colorectal cancer-associated enzyme GalNAc-T12 reveals how

nonconserved residues dictate its function. *Proc. Natl. Acad. Sci. U.S.A.* **116**, 20404–20410 (2019).

38. M. de Las Rivas, E. J. Paul Daniel, Y. Narimatsu, I. Companon, K. Kato, P. Hermosilla, A. Thureau, L. Ceballos-Laita, H. Coelho, P. Bernado, F. Marcelo, L. Hansen, R. Maeda, A. Lostao, F. Corzana, H. Clausen, T. A. Gerken, R. Hurtado-Guerrero, Molecular basis for fibroblast growth factor 23 O-glycosylation by GalNAc-T3. *Nat. Chem. Biol.* **16**, 351–360 (2020).

39. M. de Las Rivas, E. Lira-Navarrete, E. J. P. Daniel, I. Companon, H. Coelho, A. Diniz, J. Jimenez-Barbero, J. M. Peregrina, H. Clausen, F. Corzana, F. Marcelo, G. Jimenez-Oses, T. A. Gerken, R. Hurtado-Guerrero, The interdomain flexible linker of the polypeptide GalNAc transferases dictates their long-range glycosylation preferences. *Nat. Commun.* **8**, 1959 (2017).

40. T. A. Fritz, J. H. Hurley, L. Trinh, J. Shiloach, L. A. Tabak, The beginnings of mucin biosynthesis: The crystal structure of UDP-GalNAc:polypeptide α-N-acetylgalactosaminyltransferase-T1. *Proc. Natl. Acad. Sci. U.S.A.* **101**, 15307–15312 (2004).

41. H. Coelho, M. L. Rivas, A. S. Grosso, A. Diniz, C. O. Soares, R. A. Francisco, J. S. Dias, I. Companon, L. Sun, Y. Narimatsu, S. Y. Vakhrushev, H. Clausen, E. J. Cabrita, J. Jimenez-Barbero, F. Corzana, R. Hurtado-Guerrero, F. Marcelo, Atomic and specificity details of mucin 1 O-glycosylation process by multiple polypeptide GalNAc-transferase isoforms unveiled by NMR and molecular modeling. *JACS Au* **2**, 631–645 (2022).

42. F. Hanisch, S. Muller, H. Hassan, H. Clausen, N. Zachara, A. A. Gooley, H. Paulsen, K. Alving, J. Peter-Katalinic, Dynamic epigenetic regulation of initial O-glycosylation by UDP N-acetylgalactosamine:peptide N-acetylgalactosaminyltransferases. *J. Biol. Chem.* **274**, 9946–9954 (1999).

43. L. Kirnarsky, M. Nomoto, Y. Ikematsu, H. Hassan, E. P. Bennett, R. L. Cerny, H. Clausen, M. A. Hollingsworth, S. Sherman, Structural analysis of peptide substrates for mucin-type O-glycosylation. *Biochemistry* **37**, 12811–12817 (1998).

44. T. R. E. Stadie, W. Chai, A. M. Lawson, P. G. H. Byfield, F. Hanisch, Studies on the order and site specificity of GalNAc transfer to MUC1 tandem repeats by UDP-GalNAc: Polypeptide N-acetylgalactosaminyltransferase from milk or mammary carcinoma cells. *Eur. J. Biochem.* **229**, 140–147 (1995).

45. H. H. Wandall, H. Hassan, E. Mirgorodskaya, A. K. Kristensen, P. Roepstorff, E. P. Bennett, P. A. Nielsen, M. A. Hollingsworth, J. Burchell, J. Taylor-Papadimitriou, H. Clausen, Substrate specificities of three members of the human UDP-N-acetyl-α-D-galactosamine: Polypeptide N-acetylgalactosaminyltransferase family, GalNAc-T1, -T2, and -T3 *J. Biol. Chem.* **272**, 23503–23514 (1997).

46. W. F. Zeng, W. Q. Cao, M. Q. Liu, S. M. He, P. Y. Yang, Precise, fast and comprehensive analysis of intact glycopeptides and modified glycans with pGlyco3. *Nat. Methods* **18**, 1515–1523 (2021).

47. T. A. Fritz, J. Raman, L. A. Tabak, Dynamic association between the catalytic and lectin domains of human UDP-GalNAc:Polypeptide alpha-N-acetylgalactosaminyltransferase-2. *J. Biol. Chem.* **281**, 8613–8619 (2006).

48. Y. Zhang, H. Iwasaki, H. Wang, T. Kudo, T. B. Kalka, T. Hennet, T. Kubota, L. Cheng, N. Inaba, M. Gotoh, A. Togayachi, J. Guo, H. Hisatomi, K. Nakajima, S. Nishihara, M. Nakamura, J. D. Marth, H. Narimatsu, Cloning and characterization of a new human UDP-N-acetyl-alpha-D-galactosamine:polypeptide N-acetylgalactosaminyltransferase, designated pp-GalNAc-T13, that is specifically expressed in neurons and synthesizes GalNAc alpha-serine/threonine antigen. *J. Biol. Chem.* **278**, 573–584 (2003).

49. M. de Las Rivas, E. J. Paul Daniel, H. Coelho, E. Lira-Navarrete, L. Raich, I. Companon, A. Diniz, L. Lagartera, J. Jimenez-Barbero, H. Clausen, C. Rovira, F. Marcelo, F. Corzana, T. A. Gerken, R. Hurtado-Guerrero, Structural and mechanistic insights into the catalytic-domain-mediated short-range glycosylation preferences of GalNAc-T4. *ACS Cent. Sci.* **4**, 1274–1290 (2018).

50. T. Kubota, T. Shiba, S. Sugioka, S. Furukawa, H. Sawaki, R. Kato, S. Wakatsuki, H. Narimatsu, Structural basis of carbohydrate transfer activity by human UDP-GalNAc: Polypeptide alpha-N-acetylgalactosaminyltransferase (pp-GalNAc-T10). *J. Mol. Biol.* **359**, 708–727 (2006).

51. F. K. Hagen, B. Hazes, R. Raffo, D. deSa, L. A. Tabak, Structure-function analysis of the UDP-N-acetyl-d-galactosamine:polypeptide *N*-acetylgalactosaminyltransferase. *J. Biol. Chem.* **274**, 6797–6803 (1999).

52. M. R. Pratt, H. C. Hang, K. G. Ten Hagen, J. Rarick, T. A. Gerken, L. A. Tabak, C. R. Bertozzi, Deconvoluting the functions of polypeptide N-alpha-acetylgalactosaminyltransferase family members by glycopeptide substrate profiling. *Chem. Biol.* **11**, 1009–1016 (2004).

53. Z. Otwinowski, W. Minor, ″Processing of x-ray diffraction data collected in oscillation mode″ in *Methods in Enzymology*, vol. 276 of *Macromolecular Crystallography, part A*, C. W. Carter Jr., R. M. Sweet, Eds. (Academic Press, 1997), pp. 307–326.

54. E. Potterton, P. Briggs, M. Turkenburg, E. Dodson, A graphical user interface to theCCP4 program suite. *Acta Crystallogr. D Biol. Crystallogr.* **59**, 1131–1137 (2003).

55. M. D. Winn, C. C. Ballard, K. D. Cowtan, E. J. Dodson, P. Emsley, P. R. Evans, R. M. Keegan, E. B. Krissinel, A. G. W. Leslie, A. McCoy, S. J. McNicholas, G. N. Murshudov, N. S. Pannu, E. A. Potterton, H. R. Powell, R. R. Read, A. Vagin, K. S. Wilson, Overview of the CCP4 suite and current developments. *Acta Crystallogr. D Biol. Crystallogr.* **67**, 235–242 (2011).

56. P. D. Adams, P. V. Afonine, G. Bunkóczi, V. B. Chen, I. W. Davis, N. Echols, J. J. Headd, L. W. Hung, G. J. Kapral, R. W. Grosse-Kunstleve, A. J. McCoy, N. W. Moriarty, R. Oeffner, R. J. Read, D. C. Richardson, J. S. Richardson, T. C. Terwilliger, P. H. Zwart, PHENIX: A comprehensive Python-based system for macromolecular structure solution. *Acta Crystallogr. D Biol. Crystallogr.* **66**, 213–221 (2010).

57. P. Emsley, B. Lohkamp, W. G. Scott, K. D. Cowtan, Features and development of Coot. *Acta Crystallogr. D Biol. Crystallogr.* **66**, 486–501 (2010).

58. J. Jumper, R. Evans, A. Pritzel, T. Green, M. Figurnov, O. Ronneberger, K. Tunyasuvunakool, R. Bates, A. Zidek, A. Potapenko, A. Bridgland, C. Meyer, S. A. A. Kohl, A. J. Ballard, A. Cowie, B. Romera-Paredes, S. Nikolov, R. Jain, J. Adler, T. Back, S. Petersen, D. Reiman, E. Clancy, M. Zielinski, M. Steinegger, M. Pacholska, T. Berghammer, S. Bodenstein, D. Silver, O. Vinyals, A. W. Senior, K. Kavukcuoglu, P. Kohli, D. Hassabis, Highly accurate protein structure prediction with AlphaFold. *Nature* **596**, 583–589 (2021).

59. A. Morin, B. Eisenbraun, J. Key, P. C. Sanschagrin, M. A. Timony, M. Ottaviano, P. Sliz, Collaboration gets the most out of software. *eLife* **2**, e01456 (2013).

60. C. Yu, L. Liang, Y. Yin, Structural basis of carbohydrate transfer activity of UDP-GalNAc: Polypeptide N-acetylgalactosaminyltransferase 7. *Biochem. Biophys. Res. Commun.* **510**, 266–271 (2019).
