# Supplementary material for: An unusual dual sugar-binding lectin domain controls the substrate specificity of a mucin-type O-glycosyltransferase
Source: Sci Adv. 2024 Feb 28;10(9):eadj8829. doi: 10.1126/sciadv.adj8829 (PMC10901373; doi:10.1126/sciadv.adj8829)
Supplement: Supplementary file 2 — Data S1 to S3 [file sciadv.adj8829_data_s1_to_s3.zip › adj8829_Data_S1 to S3/Data_S2.gz!/T1-Muc1_modeling-and-simulations/long_distance_cc.pdf]

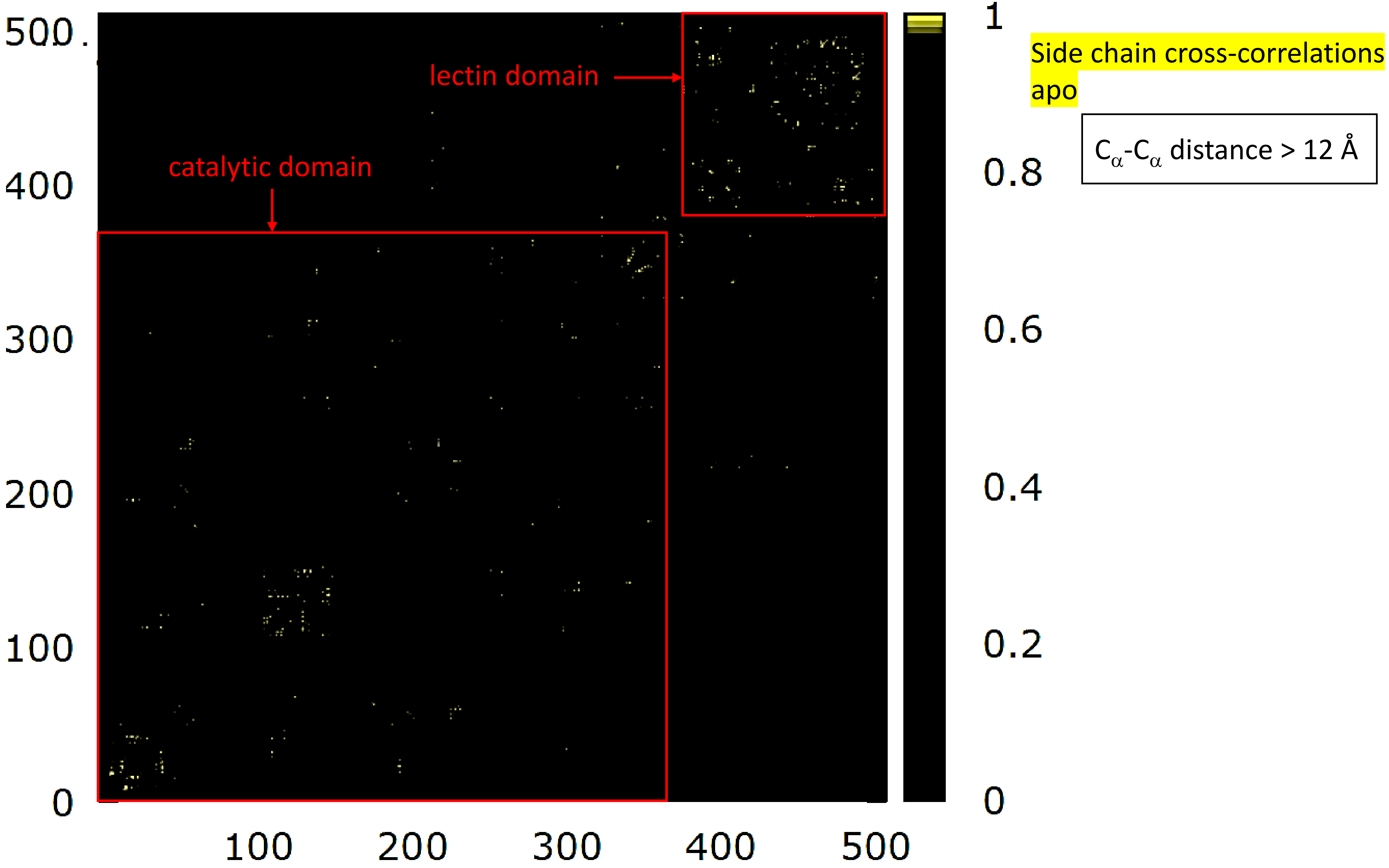

500
400
300
200
100
0
100
200
300
400
500
lectin domain
catalytic domain
1
0.8
0.6
0.4
0.2
0
Side chain cross-correlations apo
C_α-C_α distance > 12 Å

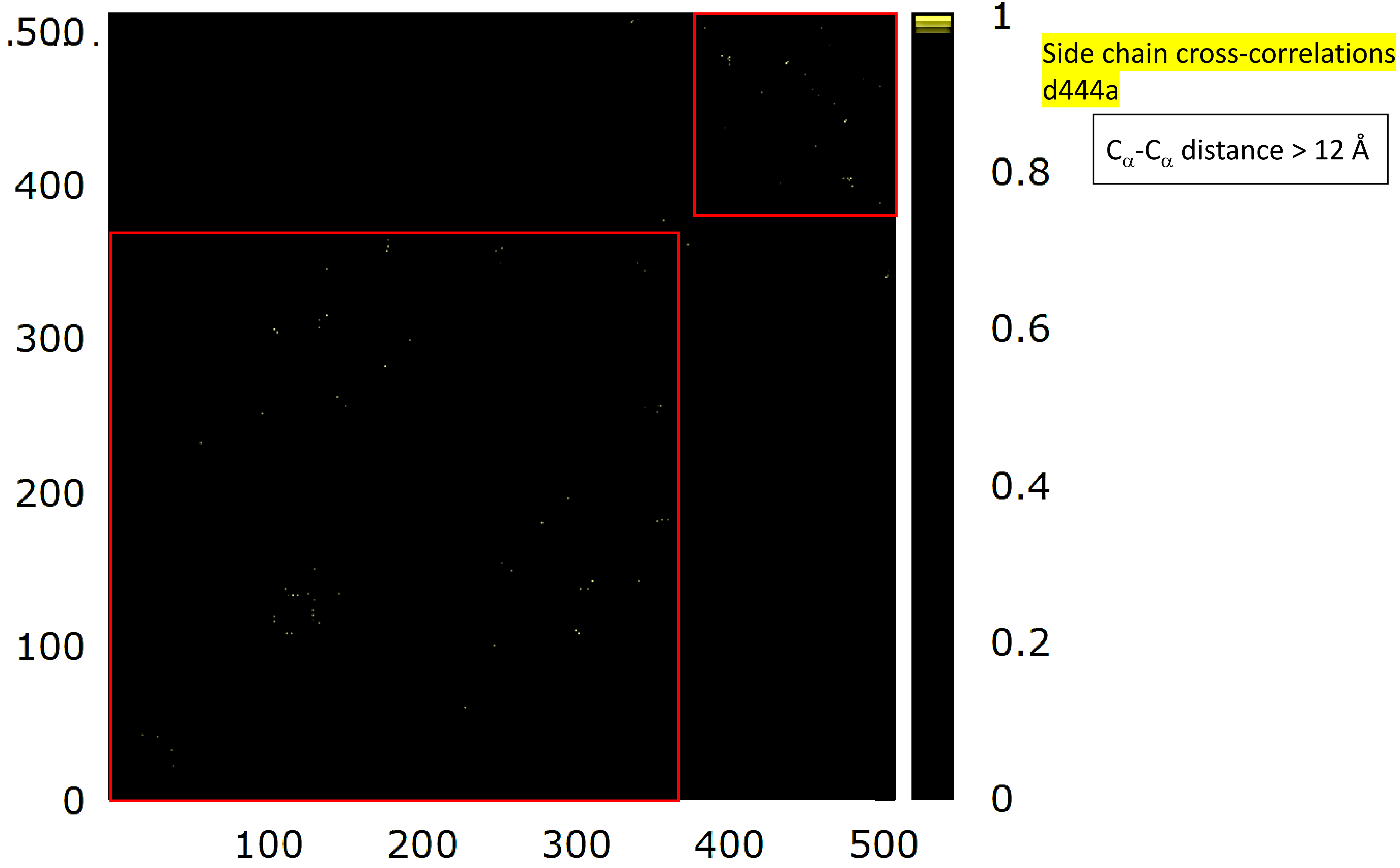

Side chain cross-correlations
d444a
C_α-C_α distance > 12 Å
500
400
300
200
100
0
100
200
300
400
500
1
0.8
0.6
0.4
0.2
0

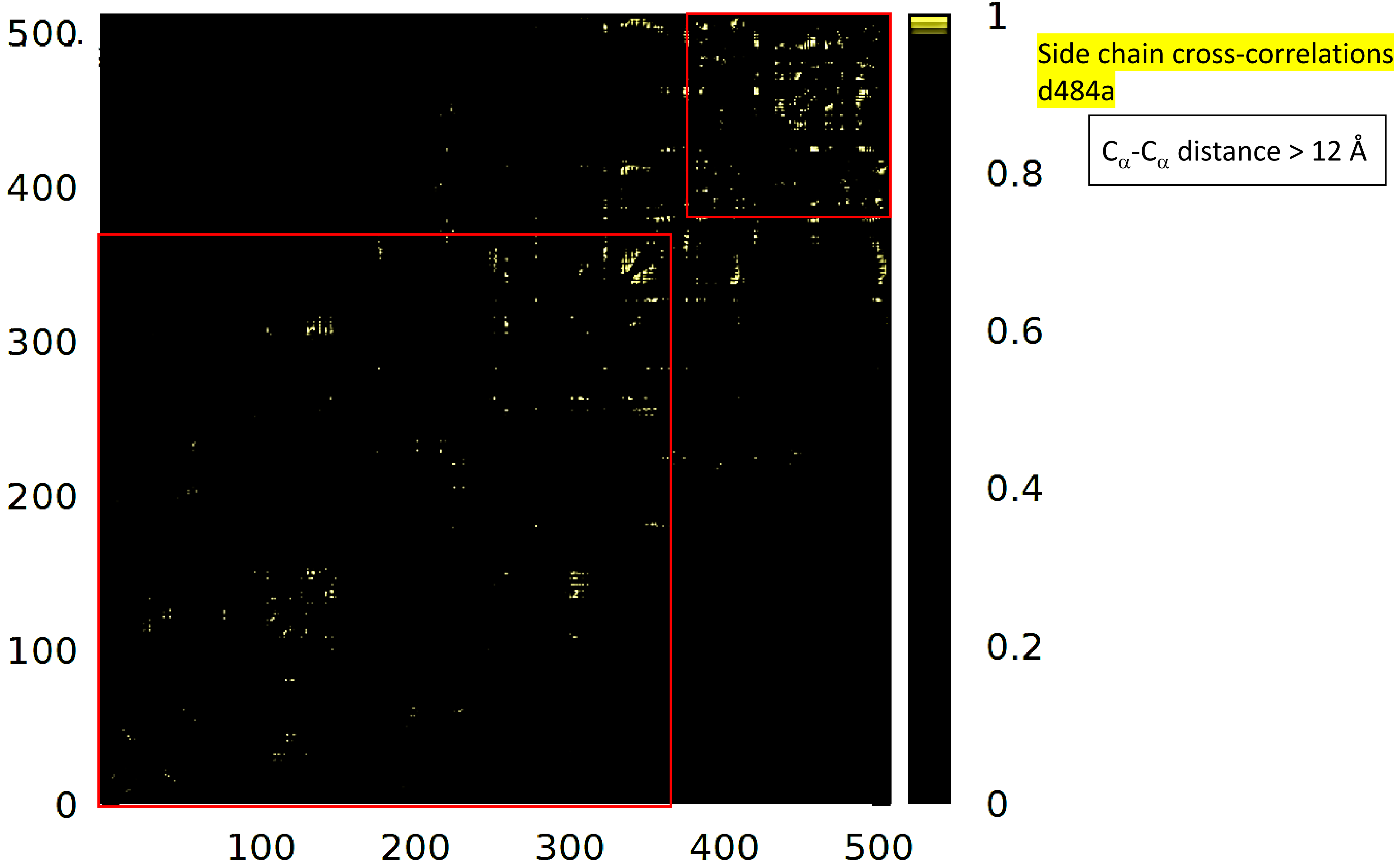

500
400
300
200
100
0
100
200
300
400
500
1
0.8
0.6
0.4
0.2
0
Side chain cross-correlations d484a
C_α-C_α distance > 12 Å

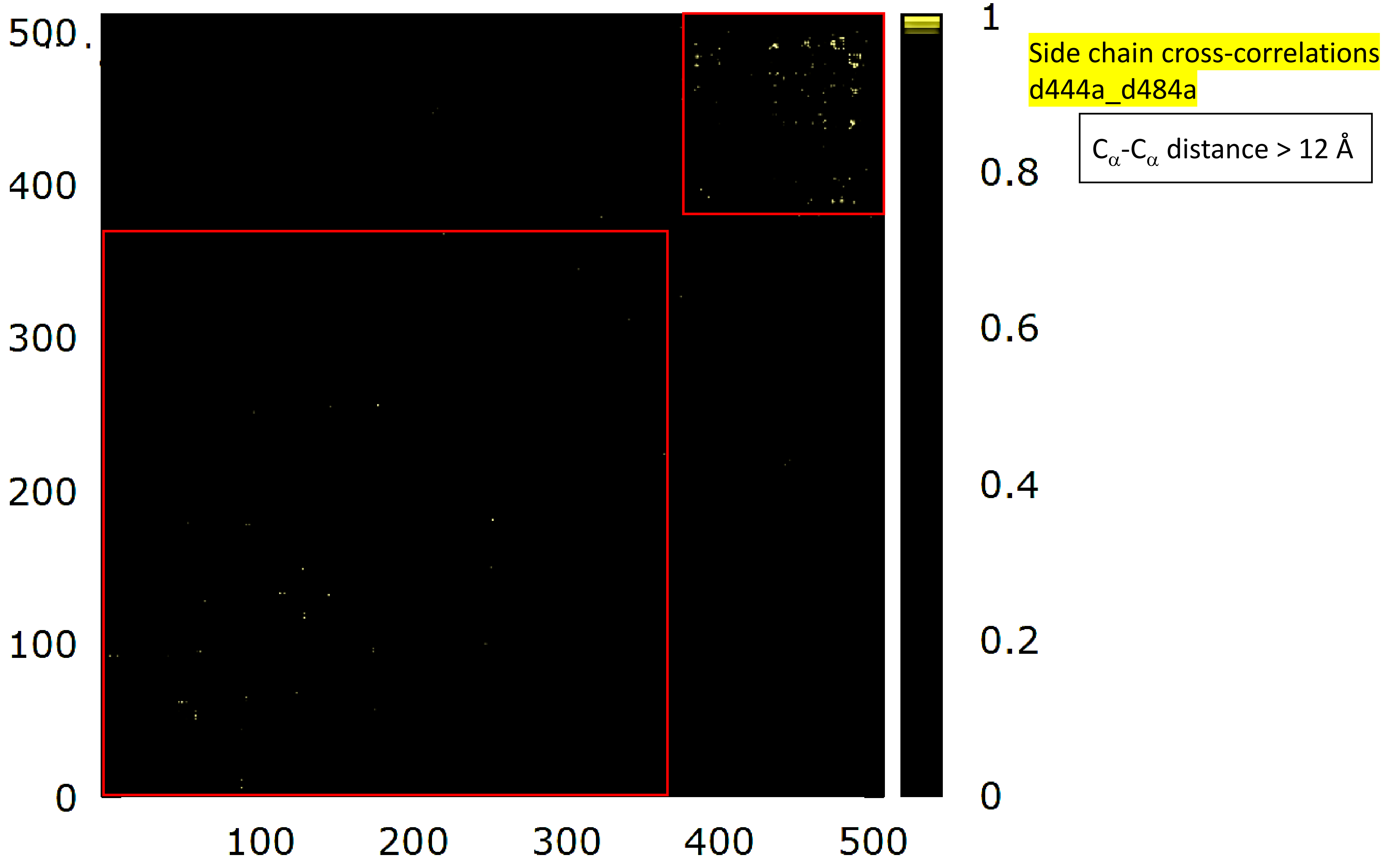

500
400
300
200
100
0
100
200
300
400
500
1
0.8
0.6
0.4
0.2
0
Side chain cross-correlations
d444a_d484a
C_α-C_α distance > 12 Å
